# Tin(IV)Porphyrin-Based Porous Coordination Polymers as Efficient Visible Light Photocatalyst for Wastewater Remediation

**DOI:** 10.3390/nano15010059

**Published:** 2025-01-02

**Authors:** Nirmal Kumar Shee, Hee-Joon Kim

**Affiliations:** Department of Chemistry and Bioscience, Kumoh National Institute of Technology, Gumi 39177, Republic of Korea; nirmalshee@gmail.com

**Keywords:** Sn(IV)porphyrin, carboxylic acid ligand, porous coordination polymers, photocatalyst, water remediation

## Abstract

Two porphyrin-based polymeric frameworks, SnP-BTC and SnP-BTB, as visible light photocatalysts for wastewater remediation were prepared by the solvothermal reaction of *trans*-dihydroxo-[5,15,10,20-tetrakis(phenyl)porphyrinato]tin(IV) (SnP) with 1,3,5-benzenetricarboxylic acid (H_3_BTC) and 1,3,5-tris(4-carboxyphenyl)benzene (H_3_BTB), respectively. The strong bond between the carboxylic acid group of H_3_BTC and H_3_BTB with the axial hydroxyl moiety of SnP leads to the formation of highly stable polymeric architectures. Incorporating the carboxylic acid group onto the surface of SnP changes the conformational frameworks as well as produces rigid structural transformation that includes permanent porosity, good thermodynamic stability, interesting morphology, and excellent photocatalytic degradation activity against AM dye and TC antibiotic under visible light irradiation. The photocatalytic degradation activities of AM dye were found to be 95% by SnP-BTB and 87% by SnP-BTC within 80 min. Within 60 min of visible light exposure, the photocatalytic degradation activities of TC antibiotic were found to be 70% by SnP-BTB and 60% by SnP-BTC. The enhanced catalytic photodegradation performances of SnP-BTB and SnP-BTC were attributed to the synergistic effect between SnP and carboxylic acid groups. The carboxylic acid connectors strongly resist the separation of SnP from the surface of SnP-BTB and SnP-BTC during the photodegradation experiments. Therefore, the high degradation rate and low catalyst loading make SnP-BTB or SnP-BTC more efficient than other reported catalysts. Thus, the present investigations on the porphyrin-based photocatalysts hold great promise in tackling the treatment of dyeing wastewater.

## 1. Introduction

Over the last two decades, soft microporous and mesoporous materials, such as porous coordination polymers (PCPs) [1,2], porous organic polymers (POPs) [3,4], metal-organic frameworks (MOFs) [5,6], and covalent organic frameworks (COFs) [7,8] have attracted significant attention owing to their interesting properties. These materials exhibit outstanding features, including high thermal and chemical stabilities, controllable permanent porosity, securing several active sites, insoluble backbones, and several functionalities. These materials are custom-designed through molecular engineering, making them an excellent alternative to conventional porous materials such as metal oxides [9], mesoporous silica [10], ceramics [11], zeolites [12], and activated charcoal [13]. These materials have been used extensively in heterogeneous catalysis [14], gas storage and separation [15], N_2_ fixation [16], hydrogen production [17], CO_2_ reduction [18], O_2_ evolution reaction [19], sensor [20], biomedicine [21], and wastewater treatment [22]. Porous coordination polymers (PCPs) are a special type of porous materials constructed by coordinating metal ions with organic building blocks in periodic two-dimensional or three-dimensional network structures. The high porosity of PCPs not only enhances catalytic activity by improving the number of catalytically active accessible sites but also facilitates the fast diffusion of substrate molecules toward the active sites, thereby enhancing the reaction rate [23,24,25]. Among the various building blocks used in the fabrication of porous coordination polymer materials, porphyrins are widely used because they transform building blocks into well-defined frameworks with uniform pores [26,27,28]. Porphyrins exhibit excellent light-harvesting properties in the solar spectrum. This is due to conjugated π electrons over the porphyrin compounds. They also exhibit excellent electron-transfer properties and are extensively used in catalysis. Monomeric porphyrins are self-assembled into nano and microstructures via supramolecular self-assembled non-covalent interactions such as hydrogen bonding interactions, π−π stacking interaction, hydrophobic or hydrophilic interactions, and van der Waals interactions [29,30,31]. Moreover, because of their inherently rigid framework and tunable porosities, the active sites are easily controllable, which promotes an understanding of their properties and structural relationships. Generally, porphyrin-based nanosize frameworks exhibit unique electronic and optical characteristics depending on the dimensions of the photofunctional materials [32,33,34,35]. In 2020, Kim et al. fabricated several porphyrin-based nanomaterials with controlled sizes and shapes [32,33]. Porphyrin-based nanofibers derived from the metal-ligand coordination are employed to degrade methylene blue (MB) dye in water under visible light irradiation [32]. Additionally, porphyrin-based ionic self-assembled nanostructures have received considerable attention for the removal of malachite green (MG) dye from water under visible light irradiation [33].

In this context, we have presented the construction, characterization, and photocatalytic application of two tin(IV)porphyrin-based porous coordination polymers. Tin(IV)porphyrin (SnP) was selected as the source of the porphyrin complex over other metalloporphyrins. This is because tin(IV)porphyrin complexes exhibit remarkable octahedral geometry and excellent optical properties. Owing to the oxophilic nature of the tin(IV) center, tin(IV)porphyrin can readily form stable six-coordinate complexes with two oxyanions, carboxylates, and alkoxides, at the *trans*-axial position. These distinctive properties of tin(IV)porphyrins make them ideal building units for the construction of multi-porphyrin arrays [36,37,38], coordination complexes [39,40,41], and nanostructures [42,43,44] with attractive properties and several utilizations. In particular, tin(IV)porphyrin-based nanomaterials have been extensively used as visible-light photocatalysts for wastewater remediation. Carboxyl acid group-based aromatic compounds were chosen as sources of organic linkers. Carboxyl acid group-based organic linkers are common in fabricating several interesting porous frameworks [45,46,47,48].

The catalytic activity of the as-prepared materials was analyzed towards visible-light catalytic photodegradation of an aqueous solution of amaranth (AM) and tetracycline (TC). AM is a highly water-soluble anionic azo dye. It is extensively used in the food, medicinal, textile, and cosmetic industries. Due to the inappropriate treatment of this azo dye in industries, AM dye may be discharged into water bodies, harming human health, and its use has been restricted in some developing countries. Additionally, AM is mutagenic and carcinogenic even at very low concentrations and is non-biodegradable. Owing to their toxic properties and high solubility in water, AM dyes are relatively difficult to remove using common physical, chemical, or biological processes. Therefore, researchers need to check the catalytic activity of as-prepared catalysts to remove AM contaminants from wastewater [49,50,51,52,53,54,55,56,57,58]. On the other hand, TC is a readily available cheap antibiotic and has broad-spectrum antimicrobial activity. It is used extensively to treat malaria, cholera, plague, brucellosis, pneumonia, typhus, and syphilis. It is also used to treat chlamydia, Rickettsia infections, intracellular mycoplasma, and Gram-positive and Gram-negative bacteria and as a growth promoter in animals. It is one of the finest and most effective medicines and has been placed on the World Health Organization’s list of essential medicines. TC belongs to the phenanthrene core group of antibiotics and is highly water-soluble. In addition, it exhibits a poor metabolic degradation rate. Therefore, the discharge of residues of this antibiotic and its metabolites into water bodies contributes to an increase in antibiotic-resistance genes in aquatic life. Owing to the high presence of this antibiotic, clean water has become sparse for humans. It has a notable effect on the environment. The most common side effects include vomiting, diarrhea, loss of appetite, rash, poor dental development, and kidney problems. Therefore, TC can be utilized as a model contaminant to determine the activity of the as-prepared catalyst [59,60,61,62,63,64,65,66,67,68,69,70,71,72].

In this regard, we aim at the fabrication of nanostructures, the morphology control, and photocatalytic performances. In particular, current interest is focused on nanomaterials based on supramolecular engineering approaches to control various structural transformations, which feature well-defined dimensions, unique porosity, high thermodynamic stability and specific morphology, and are applied to the photocatalytic degradation of pollutant dyes. For this purpose, two porphyrin-based polymeric frameworks, SnP-BTC and SnP-BTB, were fabricated from the reaction of SnP with H_3_BTC and H_3_BTB, respectively. These materials exhibited visible light catalytic photodegradation activity towards AM dye and TC antibiotic in water. A series of investigations were performed to verify the optimal conditions related to the catalytic photodegradation of AM and TC pollutants under visible light irradiation. Moreover, the kinetics and the mechanism of the photodegradation process have also been analyzed in this study. The discussion of visible-light photodegradation studies in this report is limited to AM azo dyes and TC antibiotics. However, we believe that the continued development of novel porphyrin-based porous nanostructures in conjunction with the high harvesting efficiency of visible light could also be key to the removal of water-soluble toxic metals such as Cr(VI) [73], trinitrophenol [74], and nerve agent simulant such as diethyl methylphosphonate [75].

## 2. Materials and Methods

Unless otherwise mentioned, all chemicals were procured from TCI (Tokyo Chemical Industry) and used without further purification. Particularly, 1,3,5-benzenetricarboxylic acid (H_3_BTC), 1,3,5-tris(4-carboxyphenyl)benzene (H_3_BTB), stannous chloride dihydrate (SnCl_2_∙2H_2_O). Pyrrole and toluene were distilled in calcium hydride solution. *trans*-Dihydroxo-[5,15,10,20-tetrakis(phenyl)porphyrinato]tin(IV) (SnP) was prepared following a previously reported method [76]. Elemental analysis was performed using a ThermoQuest EA 1110 analyzer. Fourier transform infrared (FT-IR) spectra (KBr) were obtained using a Shimadzu FTIR-8400S spectrophotometer (Shimadzu, Tokyo, Japan). Powder X-ray diffraction (PXRD) patterns were obtained using a Bruker AXS D8 Advance powder X-ray diffractometer (Billerica, MA, USA). Steady-state UV-vis spectra were recorded using a Shimadzu UV-3600 spectrophotometer (Shimadzu, Tokyo, Japan). The fluorescence spectra were recorded using a Shimadzu RF-5301PC fluorescence spectrophotometer (Shimadzu, Tokyo, Japan). Thermogravimetric analysis (TGA) was performed using an Auto-TGA Q500 instrument (TA Instruments, New Castle, DE, USA). Field-emission scanning electron microscopy (FE-SEM) images were obtained using a MAIA III (TESCAN, Brno, Czech Republic) instrument. The Brunauer–Emmett–Teller (BET) surface areas were measured using BELSORP-mini volumetric adsorption equipment (Micromeritics, Norcross, GA, USA) with N_2_ adsorption isotherms at 77 K. Surface and pore size data were obtained using Autosorb-iQ and Quadrasorb SI.

### 2.1. Synthesis of SnP-BTC

SnP (0.115 g, 0.15 mmol) was added to a solution of H_3_BTC (0.021 g, 0.10 mmol) dissolved in toluene (10 mL) with constant stirring under the Ar atmosphere. The reaction mixture was refluxed for 72 h in the dark. Subsequently, the reaction mixture was cooled to room temperature, and *n*-hexane (20 mL) was added and stirred for 2 h. The brown-red precipitate was filtered, washed with toluene, and air-dried. Yield: 100 mg (77%). Anal Calcd. for {C_150_H_90_N_12_O_12_Sn_3_}_n_: C, 69.07; H, 3.48; N, 6.44; R, 21.01. Found: C, 68.74; H, 3.72; N, 6.29; R, 21.25. FT-IR (KBr pellet, ν/cm^−1^): 3390 (br), 3052 (w), 2980 (w), 2565 (w), 1713 (s), 1657 (vs), 1597 (w), 1472 (w), 1442 (s), 1390 (w), 1344 (w), 1270 (vs), 1235 (w), 1215 (w), 1175 (w), 1070 (s), 1020 (vs), 922 (w), 798 (vs), 750 (vs), 705 (vs), 655 (w), 540 (s). UV-vis (BaSO_4_, *λ*_max_ (nm)): 425, 521, 562, 599, 688. Emission (Nujol, *λ*_max_ (nm)): 640, 675.

### 2.2. Synthesis of SnP-BTB

SnP (0.115 g, 0.15 mmol) was added to a solution of H_3_BTB (0.044 g, 0.10 mmol) dissolved in toluene (10 mL) with constant stirring under the Ar atmosphere. The reaction mixture was refluxed for 72 h in the dark. After that, the reaction mixture was cooled down to room temperature, and *n*-hexane (20 mL) was added and stirred for 2 h. The crimson-red precipitate was filtered, washed with toluene, and dried in air. Yield: 110 mg (72%). Anal Calcd. for {C_186_H_114_N_12_O_12_Sn_3_}_n_: C, 72.88; H, 3.75; N, 5.48; R, 17.89. Found: C, 72.67; H, 3.92; N, 5.31; R, 18.10. FT-IR (KBr pellet, ν/cm^−1^): 3405 (br), 3050 (w), 2983 (w), 2562 (w), 1712 (s), 1656 (vs), 1598 (s), 1474 (w), 1440 (s), 1388 (w), 1296 (vs), 1235 (w), 1210 (w), 1180 (w), 1072 (s), 1016 (vs), 855 (s), 798 (vs), 755 (vs), 702 (vs), 657 (w), 538 (s). UV-vis (BaSO_4_, *λ*_max_ (nm)): 431, 523, 564, 603, 688. Emission (Nujol, *λ*_max_ (nm)): 640, 675.

### 2.3. Photoelectrochemical Measurement

The photocurrent response and electrochemical impedance spectra were obtained using an electrochemical workstation (CHI660D) with 0.1 M aqueous solution of Na_2_SO_4_ as the electrolyte using a three-electrode system (a working electrode, a platinum wire electrode, and a saturated calomel electrode). The working electrode was made as follows: SnP-BTC and SnP-BTB (10 mg each) were dispersed in ethanol (5.0 mL) by sonication followed by deposition on the indium tin oxide (ITO) surface and then dried in air followed by heating at 150 °C for 12 h. Photoelectrochemical experiments have been performed under a 150 W xenon arc lamp as a visible light source.

### 2.4. Photocatalytic Degradation Experiment

The photocatalytic activities of these coordination polymers (SnP-BTC and SnP-BTB) were evaluated based on the degradation of AM dye and TC antibiotic in an aqueous solution. The photodegradation reaction was conducted under irradiation by a 150 W xenon arc lamp (ABET Technologies, Old Gate Lane Milford, CT, USA) with a UV cut-off filter (λ > 400 nm) at room temperature (298 K). In a typical procedure, 100 mg of the photocatalyst was added to a 250 mL aqueous solution of AM (100 mg L^−1^, distilled water with pH = 7.0) with stirring at 298 K. The reaction mixture was then kept in the dark for 60 min to reach an adsorption-desorption equilibrium. After irradiation with visible light, 3 mL suspension was collected at regular intervals. The catalyst was removed from the solution by centrifugation and collected through filter paper after filtration. The AM concentration was measured by determining the absorbance at 520 nm using a Shimadzu UV-3600 spectrophotometer (Shimadzu, Tokyo, Japan). On the other hand, TC solutions have been prepared from tetracycline hydrochloride with distilled water (100 mg L^−1^, pH 6.0). The exact amount of TC in the solution was estimated by calculating the optical absorbance at 371 nm.

## 3. Results and Discussion

### 3.1. Syntheses and Characterization

**Scheme 1:**
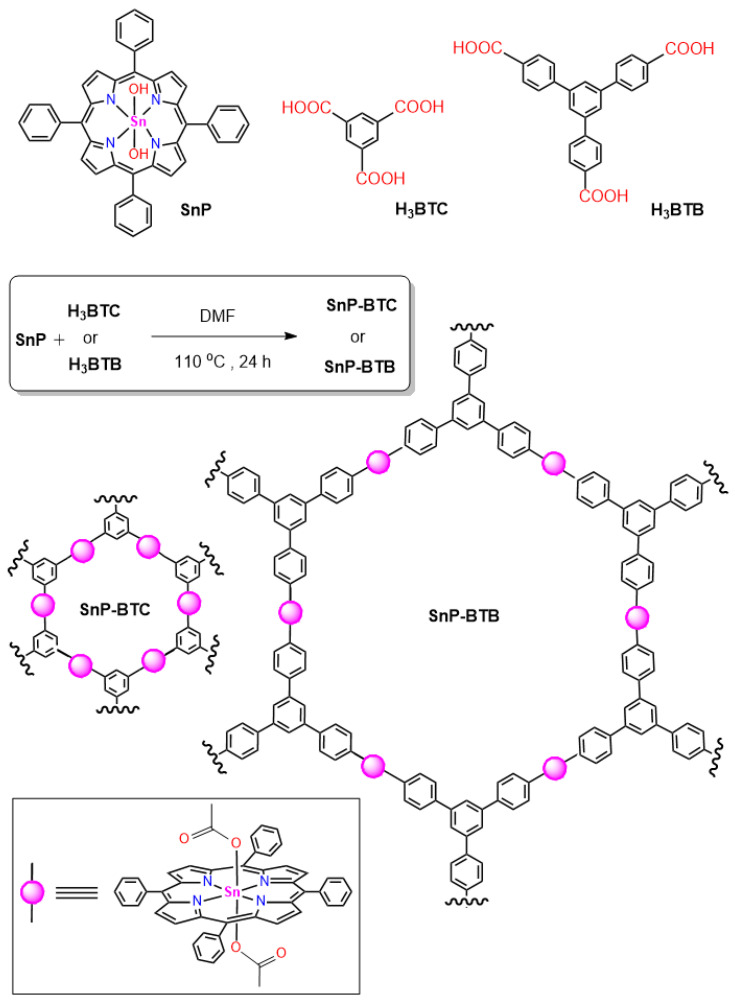
Chemical structures and synthesis of SnP-BTC and SnP-BTB.

The design and synthesis of the porphyrin-based porous frameworks (polymers (SnP-BTC and SnP-BTB) are schematically shown in Scheme 1. These materials were fabricated from the reaction of SnP with two organic linkers (H_3_BTC and H_3_BTB) under solvothermal conditions. The strong affinity of the axial hydroxyl group of the tin(IV)porphyrin towards the carboxylic acid group of H_3_BTC or H_3_BTB led to the construction of these porous architectures [39,40]. SnP-BTC and SnP-BTB were characterized using various spectroscopic techniques, including elemental analysis, UV-vis spectroscopy, emission spectroscopy, FT-IR spectroscopy, powder XRD, FE-SEM, TGA, and BET analysis.

**Figure 1:**
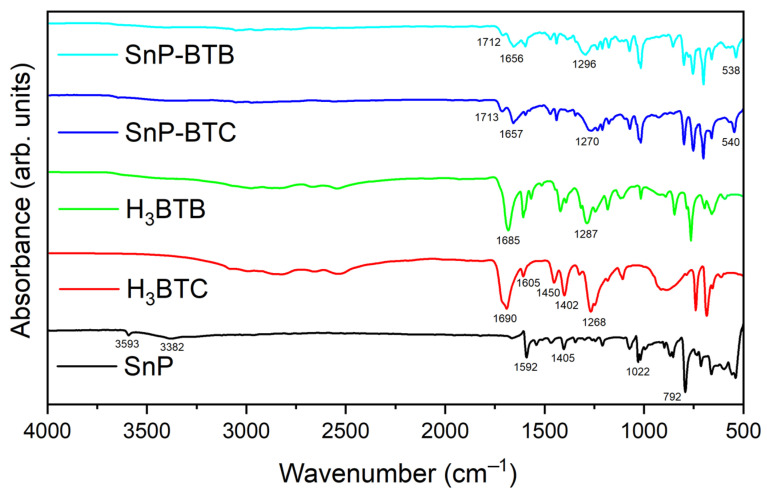
FT-IR spectra of SnP, H_3_BTC, H_3_BTB, SnP-BTC, and SnP-BTB.

The FTIR spectra of SnP, H_3_BTC, H_3_BTB, SnP-BTC, and SnP-BTB are depicted in Figure 1. In the FTIR spectrum of SnP, the peak at 3593 cm^−1^ was assigned to the stretching vibrations of the O−H signal of the axial hydroxyl group in Sn(OH)_2_TPP. The peaks at 1022 cm^−1^ and 791 cm^−1^ correspond to the bending vibration of C−H and the out-of-plane bending vibration of C−H in the aromatic ring, respectively. The peaks at 3382 cm^−1^, 1592 cm^−1^, and 1405 cm^−1^ were attributed to the stretching vibrations of C−H, C=C, and C−N in the aromatic ring, respectively. In the FTIR spectrum of H_3_BTC, the broad bands located at 2800−3100 cm^−1^ belong to the O−H stretching vibration of the carboxylic acid group and free water molecules. The broad bands at 2530−2660 cm^−1^ are assigned to the C−H stretching vibration of the aromatic ring. The strong peak at 1690 cm^−1^ was attributed to the stretching vibrations of the carbonyl group (C=O) of the aromatic carboxylic acid.

The band at 1268 cm^−1^ was assigned to the stretching vibration of C−O in the aromatic carboxylic acid. The peaks at 1605 cm^−1^, 1450 cm^−1^, and 1402 cm^−1^ were assigned to the stretching vibration of C=C in H_3_BTC. In contrast, for H_3_BTB, the characteristic peak at 1685 cm^−1^ was assigned to the C=O stretching vibration of the carboxylic acid. The peak at 1287 cm^−1^ belongs to the stretching vibration of C−O in the aromatic carboxylic acid. All other peaks were similar to those observed in the FTIR spectrum of H_3_BTC. In the FTIR spectrum of SnP-BTC, the disappearance of the peak at approximately 3590 cm^−1^ (assigned to O−H stretching in SnP) and the change in the peak position at approximately 1685 cm^−1^ (assigned to the C=O stretching in H_3_BTC) confirmed the formation of strong bonds between the hydroxyl groups of SnP and the carboxylic acid groups of H_3_BTC. The peak position of carbonyl stretching vibration appeared at 1657 cm^−1^ and 1713 cm^−1^. The peak position of C−O stretching appeared at 1270 cm^−1^ in SnP-BTC. The peak located at 540 cm^−1^ was assigned to the Sn−O stretching vibration. All the remaining peaks were similar to those of the starting materials (SnP and H_3_BTC). In the case of SnP-BTB, C=O stretching vibrations were observed at 1656 cm^−1^ and 1712 cm^−1^. A peak corresponding to C−O stretching appeared at 1296 cm^−1^. The peak located at 538 cm^−1^ was assigned to the Sn−O stretching vibration. All the remaining peaks were similar to those of the starting materials (SnP and H_3_BTB). Therefore, the disappearance of the OH stretching frequency of SnP and the peak shift of the carbonyl stretching frequency of the COOH group in SnP-BTC or SnP-BTB indicate that the coordination between the tribasic acid (H_3_BTC and H_3_BTB) and the SnP molecule is successfully formed.

**Figure 2:**
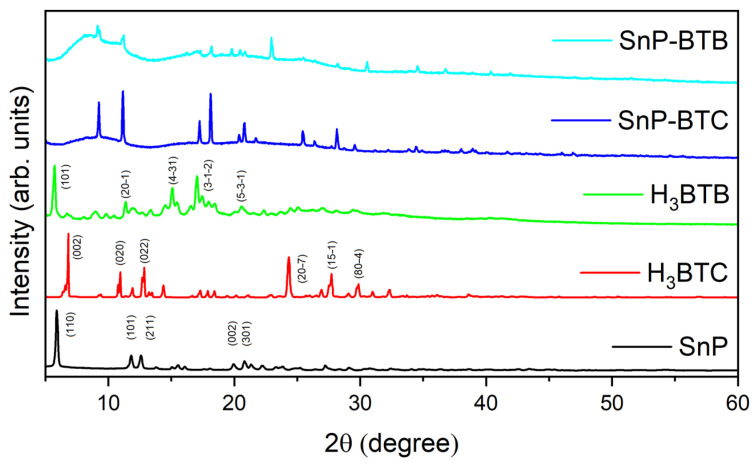
PXRD pattern of SnP, H_3_BTC, H_3_BTB, SnP-BTC, and SnP-BTB.

PXRD was used to determine the crystallinity and phase purity of the synthesized compounds. The PXRD pattern of SnP-BTC and SnP-BTB, along with the starting components (SnP, H_3_BTC, and H_3_BTB), are given in Figure 2 in the 2θ range of 5° to 60°. The diffraction peaks for the starting materials observed in the PXRD patterns were assigned based on the single crystal X-ray diffraction analysis of SnP (CCDC-1261258), H_3_BTC (CCDC-1115589), and H_3_BTB (CCDC-1008185), respectively. SnP exhibits major crystal peaks at 5.9°, 11.8°, 12.5°, 20.0°, 20.9°, and 22.2°. H_3_BTC exhibits main and sharp peaks centered at 6.8°, 10.9°, 11.9°, 12.8°, 14.4°, 24.3°, 27.8°, and 29.8°. In contrast, H_3_BTB showed the main peaks at 5.6°, 11.4°, 15.1°, 17.0°, 20.6°, and 29.5°. In the PXRD of SnP-BTC, major peaks appeared at 9.2°, 11.1°, 17.3°, 18.0°, 20.8°, 25.4°, 28.1°, and 34.5°. In contrast, SnP-BTB showed the main peaks centered at 9.2°, 11.2°, 18.2°, 23.0°, 30.5°, and 34.5°. From the PXRD patterns, it can be seen that the diffraction angle, diffraction intensity, and the distance of the crystal plane of SnP-BTC or SnP-BTB were completely different from those of SnP, H_3_BTC, and H_3_BTB. This suggests that the reaction of SnP with H_3_BTC, and H_3_BTB results in the formation of a pair of semi-crystalline complex SnP-BTC or SnP-BTB (as evident from FT-IR data). It should be noted that SnP-BTC is highly crystalline compared to SnP-BTB (Figure 2).

**Figure 3:**
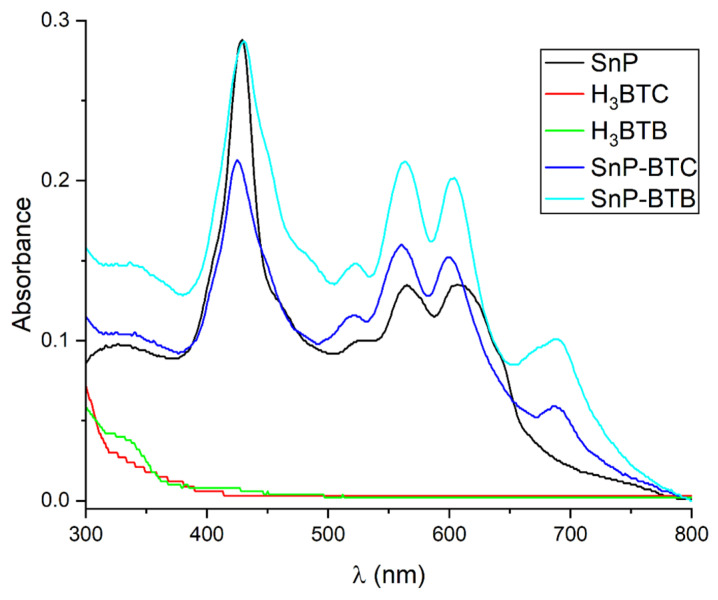
Solid state UV-vis absorption spectra of SnP, H_3_BTC, H_3_BTB, SnP-BTC, and SnP-BTB.

Solid-state UV-vis spectroscopy was conducted to examine the light-harvesting properties of SnP, H_3_BTC, H_3_BTB, SnP-BTC, and SnP-BTB (Figure 3). SnP displays a strong light absorption band centered at 429 nm assigned to the Soret band and three weak absorption peaks at 525 nm, 565 nm, and 606 nm belong to the Q bands. H_3_BTC and H_3_BTB did not exhibit characteristic optical absorbance bands in the visible region. Compared with SnP, SnP-BTC showed a strong light absorption peak at 425 nm for the Soret band and three weak peaks at 521, 562, and 599 nm for the Q band. In contrast, SnP-BTB displays a strong light absorption peak at 431 nm for the Soret band and three weak peaks at 523, 564, and 603 nm for the Q bands. The Q bands of SnP were blue-shifted in the case of SnP-BTC, whereas they were red-shifted in the case of SnP-BTB. This observation confirmed the strong attachment of SnP to SnP-BTC and SnP-BTB. An additional peak appears at approximately 688 nm for SnP-BTC and SnP-BTB, possibly due to the extended *J*-aggregation of the porphyrin rings [77,78].

**Figure 4:**
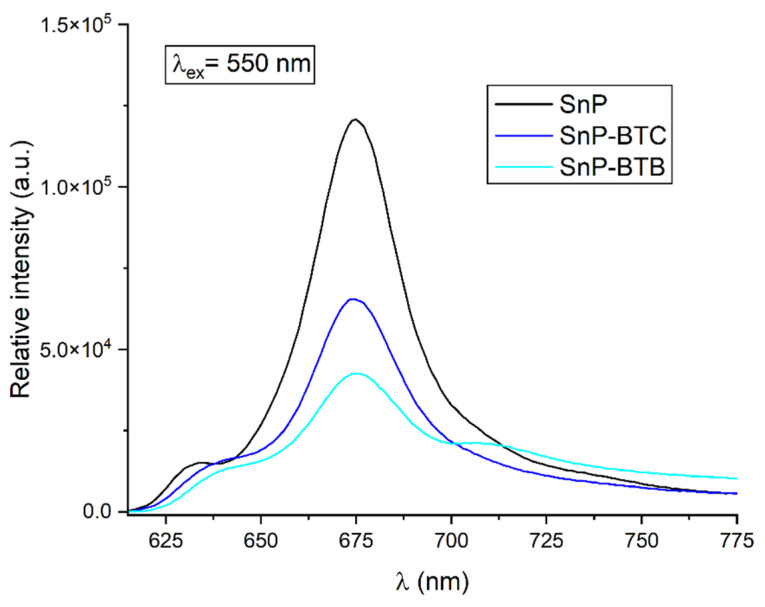
Fluorescence spectra of SnP, SnP-BTC, and SnP-BTB.

Solid-state fluorescence spectra of SnP, SnP-BTC, and SnP-BTB were recorded (Figure 4). SnP exhibits two peaks at 632 and 675 nm (λ_ex_ = 550 nm). SnP-BTC showed emission peaks at 640 and 675 nm. In contrast, SnP-BTB exhibits two peaks at 640 and 675 nm. The relative fluorescence intensities of both compounds were quenched compared to that of SnP. This observation confirms the strong attachment of SnP to the frameworks of SnP-BTC and SnP-BTB.

To study the thermal stability of SnP, SnP-BTC, and SnP-BTB, thermogravimetric analysis (TGA, Figure S1) is conducted in the temperature range from 30 to 600 °C under a flow of N_2_ with a heating rate of 5 °C min^−1^. The TG curve of SnP shows a tiny weight loss at 50–200 °C, corresponding to the removal of absorbed water molecules and organic solvents. Significant weight loss upon 370 °C is assigned to the breakdown of porphyrin moieties. In the case of SnP-BTC and SnP-BTB, a slight decrease in the curve was observed at 50–200 °C, which may be due to the absorbed solvent molecules such as water or organic solvents. Enormous weight losses were observed at around 470 °C, attributed to the decomposition of porphyrin frameworks in SnP-BTC and SnP-BTB. From these observations, it was clear that SnP-BTC and SnP-BTB possess better thermal stabilities compared to SnP.

To check the permanent porosity and porous framework of porphyrin-based materials SnP-BTC and SnP-BTB, N_2_ adsorption and desorption experiments were conducted at 77 K following a degasification at 150 °C for 10 h to remove adsorbed volatile compounds. The adsorption and desorption isotherms of N_2_ for SnP-BTC and SnP-BTB are presented in Figure S2. As depicted in Figure S2, SnP-BTC and SnP-BTB have high specific surface areas (BET) of 70.0 m^2^ g^−1^ and 190.0 m^2^ g^−1^, respectively. These materials showed type-IV adsorption-desorption isotherms, indicating their mesoporous nature. The pore width varies from ~2.1 nm to ~3.4 nm. Notably, SnP has a negligible surface area [79]. These observations confirm that SnP was successfully integrated with H_2_BTC and H_2_BTB to form porous framework materials with substantial thermal stability, high surface area, and improved nanoporous frameworks.

**Figure 5:**
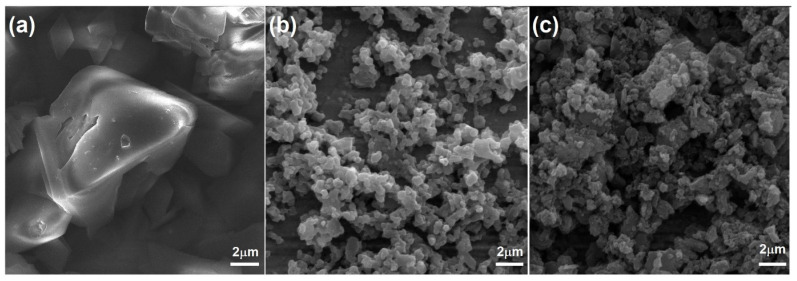
FE-SEM images for SnP (**a**), SnP-BTC (**b**), and SnP-BTB (**c**).

FE-SEM studies were conducted to inspect the self-assembly behavior, morphology, and structural development of SnP, SnP-BTC, and SnP-BTB. Each sample was suspended in H_2_O at a constant concentration (C = 0.4 mM) and centrifuged for 20 s at 13,500 rpm. After that, the solution was drop-casted on the surface of Cu-tapes, followed by drying under an oven at 80 °C. Pt-coating was required before the FE-SEM analysis. FE-SEM images of SnP, SnP-BTC, and SnP-BTB are shown in Figure 5.

The FE-SEM image shown in Figure 5a suggests that the monomeric porphyrin unit SnP did not show any exciting morphologies in the FE-SEM analysis under identical experimental conditions. In contrast, irregular nanoscopic morphologies were observed for SnP-BTC and SnP-BTB (Figure 5b and Figure 5c, respectively). The average size of these nanoparticles ranged from 600 to 900 nm. These results demonstrate that the formation of porphyrin-based covalent frameworks from the reaction of SnP with H_2_BTC and H_2_BTB leads to the fabrication of nanostructures with variable morphologies and can contribute to photocatalytic activity owing to the large surface-to-volume ratio. The elemental distributions in the porphyrin-based covalent framework materials (SnP-BTC and SnP-BTB) were examined using energy-dispersive X-ray mapping, as shown in Figures S3 and S4. The EDX data confirmed the satisfactory elemental analysis of these materials.

### 3.2. Photocatalytic Degradation of Pollutants

**Figure 6:**
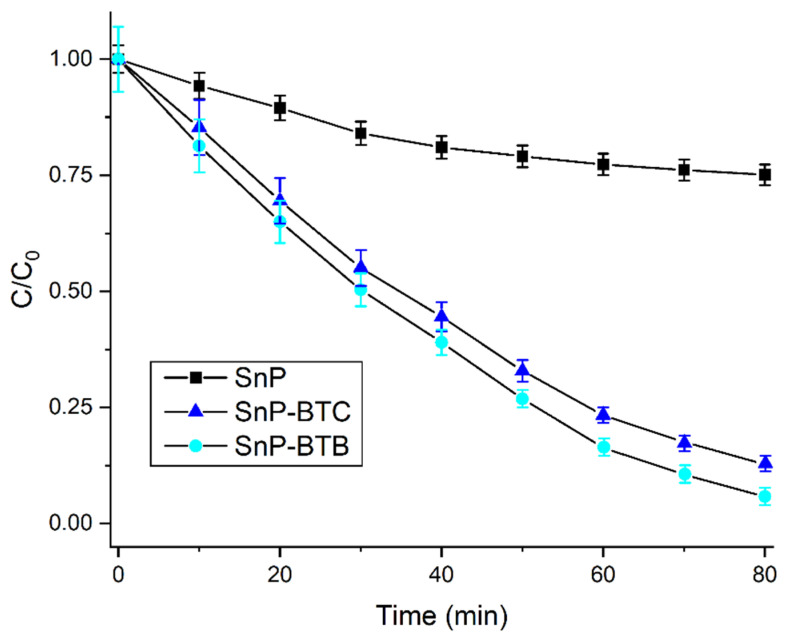
Catalytic photodegradation of AM in aqueous solution (pH 7, 298 K) under visible light irradiation.

Catalytic photodegradation of pollutants in an aqueous solution was used to evaluate the photocatalytic efficiency of the synthesized compounds under visible light irradiation. AM and TC were selected as the target pollutants for the photocatalytic degradation reactions. As shown in Figure S5, 50 min was required to attain the adsorption-desorption equilibrium. SnP, SnP-BTC absorbed 10%, and SnP-BTB absorbed 3, 10, and 19% of the AM dye. As depicted in Figure S5, SnP-BTB showed a larger uptake capacity of AM dye (35.0 mg g^−1^) than either SnP-BTC (18 mg g^−1^) or SnP (6 mg g^−1^). This confirms that the porous architectures of the porphyrin nanostructures improve the absorptivity of SnP-BTC and SnP-BTB compared to that of SnP, as well as assist mass diffusion. Time-dependent optical absorption spectra of the AM dye in the presence of SnP-BTB under visible light irradiation are shown in Figure S6. The degradation of the AM dye was observed by measuring the absorbance peak at 521 nm over a given time interval. The photodegradation performance of the various catalysts for the photodegradation of AM is expressed using the degradation efficiency (*C*_0_ − *C*)/*C*_0_, where *C*_0_ is the initial concentration of AM and *C* is the concentration at time *t*. As shown in Figure 6, SnP-BTB exhibited the highest efficiency for the photodegradation of AM within 80 min of visible-light irradiation. The calculated degradation ratios of SnP-BTB, SnP-BTC, and SnP were 95%, 87%, and 25%, respectively.

The reaction kinetics of the AM degradation were also examined. We used the pseudo-first-order model as expressed by the following equation: ln(*C*_0_/*C*) = *k*t, which is commonly used for catalytic photodegradation reactions if the initial concentration of the pollutants is low. In this equation, *k* is the pseudo-first-order rate constant. Using the data in Figure 6, we plotted ln(*C*_0_/*C*) vs. *t* and obtained the rate of AM decomposition using the synthesized catalysts (Figure S7). The first-order rate constant for the degradation of AM by SnP-BTB (0.033 min^−1^) is higher than those of SnP-BTC (0.025 min^−1^) and SnP (0.005 min^−1^). Notably, these rate constants are comparable to or even better than those previously reported for the photodegradation of AM dyes under visible light irradiation (Table 1).

**Figure 7:**
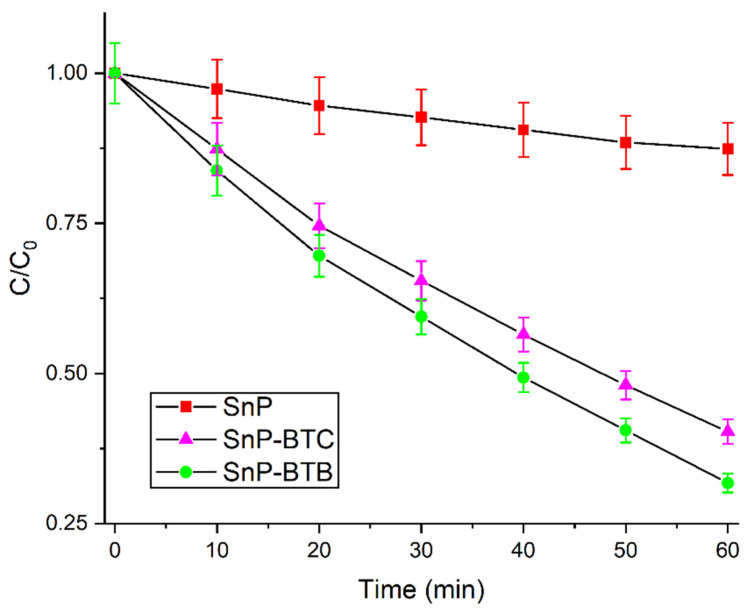
Visible light photodegradation of an aqueous solution of TC (pH 5.8, temp = 298K).

The universality of the catalytic activity of the synthesized materials was further examined for the photocatalytic photodegradation of TC. The time-dependent absorption spectra of TC for SnP-BTB are shown in Figure S8. The TC degradation was examined by measuring the absorbance peak at 371 nm at regular intervals. Within 60 min of visible-light irradiation, the calculated degradation ratios reached 70%, 60%, and 13% for the catalysts derived from SnP-BTB, SnP-BTC, and SnP, respectively (Figure 7). As shown in Figure S9, the pseudo-1st-order decay rate constant for the TC by SnP-BTB (0.019 min^−1^) is higher than either SnP-BTC (0.015 min^−1^) or SnP (0.003 min^−1^). Table 2 compares the photodegradation rate constants of TC with those of previously reported catalysts under identical conditions (Table 2).

From the above results, it is clear that SnP-BTC and SnP-BTB show significant catalytic photodegradation performance for water contaminants. The reusability of the photocatalyst is important for industrial purposes, as confirmed by recycling tests of SnP-BTB for AM dye photodegradation. As shown in Figure S10, SnP-BTB retained excellent catalytic photodegradation activity for the AM dye, with a slight decrease (~8%) after 10 consecutive cycles. The degradation activity of SnP-BTB gradually decreased with increasing recycling cycles, which might be the result of by-products deposited on the surface of the SnP-BTB catalyst. Moreover, the procedures for catalyst recovery after decomposition reactions are very simple. After the degradation reactions, the catalysts were separated by centrifugation, filtered, washed twice with distilled water, and dried under vacuum at 70 °C for 6 h. The morphologies of SnP-BTB (fresh and used samples) were evaluated using FE-SEM. From Figure S11, it is clear that there is no significant difference between the used and fresh catalysts. Additionally, the FT-IR (Figure S12) and PXRD spectra (Figure S13) of SnP-BTB confirm the high stability of the photocatalyst during the degradation reactions. These results show that the degradation performance and stability of the photocatalyst remained unchanged, making it a promising photocatalyst for wastewater treatment under visible light irradiation.

The optimum conditions for the catalytic degradation reactions must be fixed in terms of the AM dye/catalyst ratio, solution pH, and temperature. Photodegradation experiments were conducted at various temperatures to determine the effect of temperature on the dye degradation rate. The photodegradation of the AM dye gradually increased with increasing temperature (Figure S14). From Figure S15, it was confirmed that the degradation rate of the AM dye increased as the pH of the solution increased from 2 to 7. Subsequently, the degradation rate decreased with increasing solution basicity (up to pH 12). As shown in Figure S15, the degradation rate was more affected in the basic medium than in the acidic medium. The photodegradation rate is lower in the basic medium. Basic hydrolysis is likely to result in the axial bond cleavage between SnP and the carboxylate group of H_3_BTC or H_3_BTB. The degradation rate of AM dyes is slightly lower in acidic media due to the protonation of the sulfonic acid group of AM dyes. To know the effect of AM dye/SnP-BTB ratio on the catalytic photodegradation experiments, several concentrations of AM dye solution (starting from 50 to 150 mg L^−1^) have been used along with a fixed amount of SnP-BTB (400 mg L^−1^). The catalytic degradation rate of the AM dye decreased with increasing concentrations of the AM dye (Figure S16). The light did not reach the SnP-BTB surface at high AM dye concentrations.

In addition, several monochromatic lights of different wavelengths were considered to determine the effect of the light source on the photocatalytic AM dye removal rate using SnP-BTB (Figure S17). The range of irradiation wavelengths was controlled by using various optical filters. A significant variation was observed in the wavelength-dependent catalytic photodegradation of the AM dye. The wavelength of the light source likely contributes significantly to solar energy harvesting, which is followed by performance degradation. Moreover, SnP-BTB still exhibited little photocatalytic decomposition activity even at λ > 700 nm.

ESI-MS analysis was conducted to determine the complete details of AM dye degradation products during the reaction. For this purpose, 2 mL of the reaction mixture was removed after 40 min, and the mass was measured by ESI-MS (Figure S18). New peaks appearing in the mass spectra confirmed the degradation of the AM dye to lower fragments [56]. Based on Figure S18, the possible intermediates for the decomposition of the AM dye are shown in Figure S19. Initially, the base peak (*m*/*z* = 537.0) was associated with the anionic form of AM dye. AM dye can undergo asymmetric cleavage of azo bonds to form lower fragments with *m*/*z* 222.0 and *m*/*z* 318.0. Subsequently, these two aromatic intermediate species can undergo consecutive ring-opening and hydrolysis to form smaller fragments (*m*/*z* 165.0, *m*/*z* 137.0). These smaller fragments could then undergo further ring cleavage and oxidation, leading to the generation of a smaller intermediate with *m*/*z* = 117.0. Finally, all smaller fragments were further disintegrated and mineralized into low-toxicity CO_2_ and H_2_O. Additionally, the total organic carbon (TOC) value was estimated to calculate the removal of AM dye by SnP-BTB and SnP-BTC [56]. The TOC removal percentage was found to be 85% for SnP-BTB and 72% for SnP-BTC.

### 3.3. Possible Mechanism of the Photocatalytic Degradation of Contaminants

So far, SnP-BTB has shown better catalytic pollutant degradation performance than either SnP-BTC or SnP. Before discussing the possible mechanism of the catalytic degradation reaction, let us examine the reasons. The catalytic degradation activity likely depends on some important factors, such as the light-harvesting properties, specific surface area, morphology, and band gap energy (E_g_) of the catalyst. Moreover, the separation and transport of the photogenerated charge species and their recombination times are important for the degradation reaction. The band gap energy (E_g_) was calculated from the Tauc plot using the optical absorption spectra (Figure S20). The estimated E_g_ is ~2.64 eV for SnP-BTB, which is lower than that of either SnP-BTC (~2.67 eV) or SnP (~2.84 eV). Therefore, the lower band gap energy of SnP-BTB indicates that it attains a better light-harvesting ability than either SnP-BTC or SnP. As shown in Figure 4 (λ_ext_ = 550 nm), SnP-BTB shows significant quenching (~66%) of the emission intensity compared with SnP. On the other hand, SnP-BTC also shows similar emission quenching (~50%) compared to SnP. These observations indicate the restricted recombination of the photogenerated hole (h^+^) and electron (e^−^) pairs, and strong electronic delocalization happens over the surface of the conjugated aromatic moieties.

Photoelectrochemical experiments were conducted to determine the role of the charge-transfer complexes in the photocatalytic degradation of SnP-BTB, SnP-BTC, and SnP. As depicted in Figure S21, SnP-BTB shows a better photocurrent response than either SnP-BTC (1.6 times lower than that of SnP-BTB) or SnP (5.2 times lower than that of SnP-BTB). These results indicate that the photogenerated charge-carrier separation was significantly enhanced in the order SnP < SnP-BTC < SnP-BTB. Additionally, the EIS-Nyquist plot of SnP-BTB shows a lower arc radius than that of either SnP-BTC or SnP (Figure S22), indicating that the working electrode has a smaller charge transfer resistance under visible light irradiation [80]. These observations indicate that SnP-BTB is better than either SnP-BTC or SnP in terms of separation and transportation of the photogenerated reactive species.

Radical trapping tests were conducted to explore the characteristics of the photogenerated reactive species in the photodegradation experiments [81,82,83]. For this purpose, *para*-benzoquinone (*p*-BQ) was employed to trap O_2_^−•^ (superoxide radical anion), *tert*-butanol (*t*-BuOH) was used to capture ^•^OH (hydroxyl radical), ethylenediaminetetraacetic acid disodium (Na_2_-EDTA) was utilized to detect photogenerated holes (h^+^), and sodium azide (NaN_3_) was utilized to seize singlet O_2_ during the photodegradation reaction of AM dye in presence of SnP-BTB (Figure S23). In the presence of Na_2_-EDTA, *t*-BuOH, and *p*-BQ, the photodegradation performance of SnP-BTB is seriously influenced (Figure S23). However, the catalytic degradation of AM was not affected by the presence of NaN_3_ or singlet oxygen. The catalytic photodegradation removal rate of the AM dye decreased significantly in the presence of Na_2_-EDTA (~25%) compared with either *t*-BuOH (~43%) or *p*-BQ (~54%). From the above observations, it was confirmed that photogenerated holes (h^+^) were the main reactive species that catalyzed the degradation of AM dye. It should be noted that the catalytic photodegradation of the AM dye is impossible without a catalyst or visible light (Figure S23).

From the above observations, the mechanism of photodegradation of the AM dye by the porphyrin-based photocatalyst (P) has been proposed as follows [84,85,86]. After irradiation of visible light, P can be excited after crossing the band gap energy and producing sufficient hole-electron pairs (h^+^/e^−^). These excited electrons can trigger strong electronic delocalization over the surface of the conjugated aromatic frameworks in P and delay the recombination with holes. This will facilitate the generation of photogenerated charge pairs, their transportation, and separation from recombination. A highly reactive hydroxyl radical (^•^OH) is generated after the reaction of H_2_O with a photogenerated hole (h^+^), which degrades the AM dye into smaller fragments. The excited electrons can react with O_2_ to produce highly reactive superoxide radical anions (O_2_^−•^) and degrade the AM dye into lower intermediates. Finally, all the lower intermediates were further mineralized to CO_2_ and H_2_O. In summary, the reaction mechanism of a porphyrin-containing catalyst (P) comprises five steps.

T + *hν* → T^∗^(e^−^ + h^+^) (1)

O_2_ + e^−^ → O_2_^−•^ (2)

H_2_O + h^+^ → ^•^OH + H^+^ (3)

AM + O_2_^−•^ → Degraded products (4)

AM + ^•^OH → Degraded products (5)

**Figure 8:**
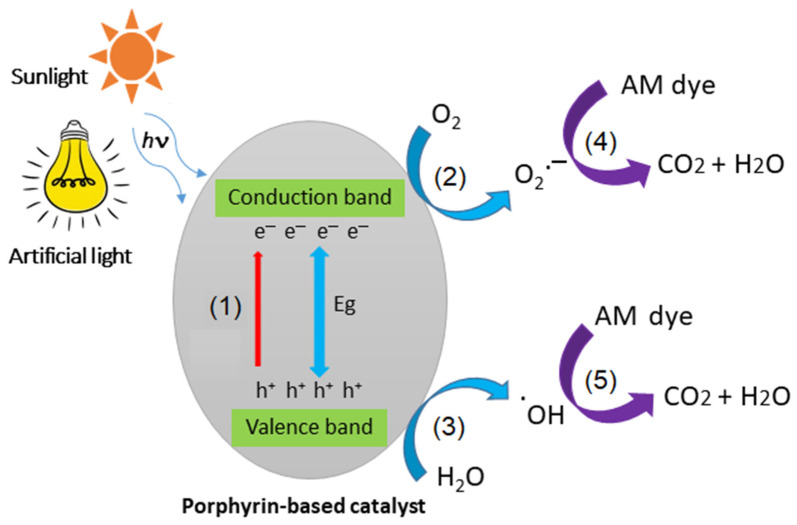
A schematic representation of the proposed mechanism for visible light photodegradation of AM dye.

In addition, the mechanism of the proposed AM dye photodegradation by the porphyrin-based photocatalyst is schematically represented in Figure 8.

Compared with the starting porphyrin SnP, the porphyrin-based framework materials SnP-BTB and SnP-BTC showed significantly enhanced photocatalytic photodegradation performance, which can be described as follows: (a) SnP-BTB and SnP-BTC can remarkably improve the light-harvesting properties in the solar light spectrum because of their lower band gap energy compared to SnP. This will produce more photogenerated reactive species to facilitate catalytic photodegradation. (b) Compared to SnP, the self-assembled structures of SnP-BTB and SnP-BTC can strongly stabilize the photogenerated reactive pairs over the aromatic conjugated frameworks, providing multidimensional channels for charge carrier transfer and separation, and hence delaying the recombination process. (c) The permanent porosity of SnP-BTB and SnP-BTC with a large active specific surface area permitted increased adsorption ability towards toxic contaminants, thereby improving the photodegradation experiment. (d) More reactive species (h^+^, ^•^OH, and O_2_^−•^) are generated in the degradation process, which can significantly enhance the photodegradation activity of porphyrin-based framework materials SnP-BTB and SnP-BTC compared to monomeric porphyrin units of SnP.

## 4. Conclusions

In summary, two porphyrin-based porous coordination frameworks (SnP-BTB and SnP-BTC) were synthesized by reacting tin(IV)porphyrin (SnP) with two aromatic carboxylic ligands, H_3_BTC and H_3_BTB. The strong bond of the carboxylic acid groups of H_3_BTC and H_3_BTB towards the axial hydroxyl moiety of SnP led to the formation of highly stable polymeric architectures. During the photodegradation experiments, the carboxylic acid connectors strongly resist the separation of SnP from the surfaces of SnP-BTB and SnP-BTC. Therefore, the chemically strong attachment between SnP and the aromatic carboxylic acid group not only increased the amount of SnP on the surface of both SnP-BTB and SnP-BTC but also stabilized the photogenerated reactive species produced during the photodegradation reaction. This delays the recombination of the photogenerated reactive species, enhancing the photocatalytic activity. This enhanced the visible-light photocatalytic degradation of toxic compounds in H_2_O. The enhanced catalytic photodegradation performances of SnP-BTB and SnP-BTC were mostly attributed to the synergistic effect between SnP and the carboxylic acid groups. Incorporating a carboxylic acid group onto the surface of SnP changes the conformational framework. It produces a rigid structural transformation that includes permanent porosity, good thermodynamic stability, interesting morphology, and excellent photocatalytic degradation activity against AM and TC antibiotics under visible-light irradiation. The photocatalytic degradation activity of AM dye was found to be 95% (*K* = 0.033 min^−1^) by SnP-BTB and 87% (*K* = 0.025 min^−1^) by SnP-BTC within 80 min. Within 60 min of visible light exposure, the photocatalytic degradation activity of TC antibiotic was found to be 70% (*K* = 0.019 min^−1^) by SnP-BTB and 60% (*K* = 0.015 min^−1^) by SnP-BTC. Therefore, the high photocatalytic degradation rate and low catalyst loading make SnP-BTB and SnP-BTC more efficient than the other reported conventional visible-light catalysts. Thus, this report holds great promise for treating dyeing wastewater using various porphyrin-based photocatalysts.

## Figures and Tables

**Table 1:** A comparison of the photodegradation efficiency of AM dye with various photocatalysts.

Photocatalysts	Irradiation Time (min)	Catalyst Dosage (mg/L)	Dye Concentration (mg/L)	Rate Constant (min^−1^)	References
TiO_2_ (P-25)	90	500	50	0.0270	[49]
H_2_O_2_-TiO_2_	300 (UV)	160	48	0.0160	[50]
Immobilized TiO_2_ film	300 (UV)	Not applicable	10	0.0098	[51]
N-doped ZnO	240	1000	10	0.0091	[52]
Photocatalytic fuel cell-electro fenton (PEC-EF)	480	Not applicable	10	0.0070	[53]
SnO_2_	120	1000	12	0.0023	[54]
BiVO_3_	120	1000	12	0.0063	[54]
BiVO_3_/SnO_2_	120	1000	12	0.0230	[54]
Sn(OH)_2_TPyP-Ag CPs	90	33	10	0.0210	[55]
Sn(INA)_2_TPyP-Ag CPs	90	33	10	0.0310	[55]
ZnO	60	200	50	0.0060	[56]
SnP@ZnO	60	200	50	0.0080	[56]
SnP/AA@ZnO	60	200	50	0.0480	[56]
g-C_3_N_4_	150	1000	10	0.0038	[57]
BiOI	150	1000	10	0.0060	[57]
g-C_3_N_4_/BiOI	150	1000	10	0.0078	[57]
Tourmaline	150	1000	10	0.0005	[57]
g-C_3_N_4_/Tourmaline	150	1000	10	0.0051	[57]
BiOI/Tourmaline	150	1000	10	0.0069	[57]
g-C_3_N_4_/BiOI/Tourmaline	150	1000	10	0.0122	[57]
g-C_3_N_4_	150	1000	10	0.0038	[57]
MgO	150	333	10	0.0110	[58]
Cu_3_(PO_4_)_2_/MgO	150	333	10	0.0220	[58]
SnP	80	400	100	0.005	This work
SnP-BTC	80	400	100	0.025	This work
SnP-BTB	80	400	100	0.033	This work

**Table 2:** A comparison of the photocatalytic decomposition activity of TC with various photocatalysts.

Photocatalysts	Irradiation Time (min)	Catalyst Dose (mg/L)	TC Concentration (mg/L)	Rate Constant (min^−1^)	References
AgIn(MoO_4_)_2_	60	1000	10	0.00698	[59]
6%-Ag/AgIn(MoO_4_)_2_	60	1000	10	0.00985	[59]
Ag_2_CO_3_/Ag/WO_3_	90	1000	10	0.0179	[60]
g-C_3_N_4_	180	1000	10	0.0032	[61]
CdIn_2_S_4_	180	1000	10	0.00422	[61]
rGO/g-C_3_N_4_	180	1000	10	0.00251	[61]
rGO/CdIn_2_S_4_	180	1000	10	0.0042	[61]
CdIn_2_S_4_/g-C_3_N_4_	180	1000	10	0.0043	[61]
rGO/30%-CdIn_2_S_4_/g-C_3_N_4_	180	1000	10	0.00766	[61]
CuBi_2_O_4_	120	500	10	0.00341	[62]
Fe_2_O_3_	120	500	10	0.00485	[62]
30%-Fe_2_O_3_/CuBi_2_O_4_	120	500	10	0.01246	[62]
polyacrylonitrile (PAN)-TiO_2_/Ag	240	1000	20	0.013	[63]
La_2-x_Sr_x_NiMnO_6_ (x = 0)	240	1000	10	0.0072	[64]
La_2-x_Sr_x_NiMnO_6_ (x = 0.10)	240	1000	10	0.0101	[64]
CuBi_2_O_4_	120	500	10	0.0032	[65]
MoS_2_	120	500	10	0.0034	[65]
CuBi_2_O_4_/MoS_2_ (5%)	120	500	10	0.0095	[65]
CdS	90	250	40	0.0056	[66]
γ-In_2_Se_3_ (0.04 M EDTA)	120	1000	20	0.0175	[67]
TiO_2_ (P-25)	150	200	10	0.0069	[68]
BP (black phosphorus)	90	1000	50	0.00068	[69]
BiOBr	90	1000	50	0.0027	[69]
10BP/BiOBr	90	1000	50	0.011	[69]
poly vinylidene fluoride-TiO_2_@g-C_3_N_4_-0.2g	300	1000	50	0.012	[70]
TiO_2_	100	250	10	0.0055	[71]
20 wt% MoS_2_/TiO_2_	100	250	10	0.0185	[71]
Bi_2_WO_6_	180	300	20	0.00438	[72]
Oxygen deficient Bi_2_WO_6_	180	300	20	0.00783	[72]
SnP	60	400	125	0.003	This work
SnP-BTC	60	400	125	0.015	This work
SnP-BTB	60	400	125	0.019	This work

## Data Availability

Data are contained within the article or Supplementary Material.

## Supplementary Materials

The following supporting information can be downloaded from https://www.mdpi.com/article/10.3390/nano15010059/s1. Figure S1. Thermograms of SnP, SnP-BTC, and SnP-BTB. Figure S2. Adsorption and desorption isotherms of N_2_ for SnP-BTC and SnP-BTB at 77 K. Figure S3. Energy-dispersive X-ray spectroscopy (EDS) of SnP-BTC. Elemental mapping (a), spectra (b), table (c). Figure S4. Energy-dispersive X-ray spectroscopy (EDS) of SnP-BTB. Elemental mapping (a), spectra (b), table (c). Figure S5. AM dye adsorption tests of SnP, SnP-BTC, and SnP-BTB. Figure S6. Absorption spectra of the AM dye in the presence of SnP-BTB under visible light irradiation. Figure S7. Kinetics of photocatalytic degradation of AM under visible light irradiation. Figure S8. Absorption spectra of TC in the presence of SnP-BTB under visible light irradiation. Figure S9. Kinetics of photocatalytic degradation of TC under visible light irradiation. Figure S10. Recyclability of the SnP-BTB photocatalyst towards the degradation of AM dye degradation. Figure S11. FE-SEM images of SnP-BTB before and after degradation of AM dye). Fresh (a) and used (b) samples. Figure S12. FT-IR spectra of SnP-BTB (before and after AM dye degradation). Figure S13. PXRD patterns of SnP-BTB (before and after degradation of the AM dye). Figure S14. Effect of temperature on the photocatalytic degradation of AM in the presence of SnP-BTB. Figure S15. Effect of pH on the degradation of AM dye solution in the presence of SnP-BTB. Figure S16. Effect of dye concentration on the photocatalytic degradation of AM in the presence of SnP-BTB. Figure S17. Effect of light intensity on the photocatalytic degradation of AM in the presence of SnP-BTB. Figure S18. Negative ion mode ESI-mass spectrum of AM dye degradation by SnP-BTB after 40 min of visible light irradiation. Figure S19. Possible intermediates of AM dye degradation in the presence of SnP-BTB after 40 min of visible light irradiation. Figure S20. The band gap energies of SnP-BTB, SnP-BTC, and SnP were calculated from Tauc’s plots using absorption spectral data. Figure S21. Photocurrent response of SnP-BTB, SnP-BTC, and SnP under visible light irradiation Figure S22. EIS Nyquist plots of SnP-BTB, SnP-BTC, and SnP under visible light irradiation. Figure S23. Visible light AM dye degradation activity of SnP-BTB in the presence of various scavengers.
