# Supplementary material for: Tin(IV)Porphyrin-Based Porous Coordination Polymers as Efficient Visible Light Photocatalyst for Wastewater Remediation
Source: Nanomaterials (Basel). 2025 Jan 2;15(1):59. doi: 10.3390/nano15010059 (PMC11722641; doi:10.3390/nano15010059)
Supplement: Supplementary file 1 [file nanomaterials-15-00059-s001.zip › nanomaterials-3368860-supplementary.pdf]

Supplementary Materials

Tin(IV)Porphyrin-Based Porous Coordination Polymers as Efficient Visible Light Photocatalyst for Wastewater Remediation

Nirmal Kumar Shee and Hee-Joon Kim*

Department of Chemistry and Bioscience, Kumoh National Institute of Technology
Gumi 39177, Republic of Korea

List of contents:

Figure S1. TGA thermogram of SnP, SnP-BTC, and SnP-BTB.

Figure S2. Adsorption and desorption isotherms of N_2 for SnP-BTC and SnP-BTB at 77 K.

Figure S3. Energy dispersive X-ray spectroscopy (EDS) of SnP-BTC. Elemental mapping (a), spectra (b), table (c).

Figure S4. Energy dispersive X-ray spectroscopy (EDS) of SnP-BTB. Elemental mapping (a), spectra (b), table (c).

Figure S5. AM dye adsorption test of SnP, SnP-BTC, and SnP-BTB.

Figure S6. Absorption spectra of AM dye in the presence of SnP-BTB under visible light irradiation.

Figure S7. Kinetics of the photocatalytic degradation of AM under visible light irradiation.

Figure S8. Absorption spectra of TC in the presence of SnP-BTB under visible light irradiation.

Figure S9. Kinetics of the photocatalytic degradation of TC under visible light irradiation.

Figure S10. Recyclability of the photocatalyst SnP-BTB towards the degradation of AM dye.

Figure S11. FE-SEM images of SnP-BTB after and before the degradation of AM dye). Fresh sample (a), and used sample (b).

Figure S12. FT-IR spectra SnP-BTB (after and before the degradation of AM dye).

Figure S13. PXRD spectra SnP-BTB (after and before the degradation of AM dye).

Figure S14. Effect of temperature for the photocatalytic degradation of AM dye in the presence of SnP-BTB.

Figure S15. Effect of pH for the degradation of AM dye solution in the presence of SnP-BTB.

Figure S16. Effect of dye concentration for the photocatalytic degradation of AM dye in the presence of SnP-BTB.

Figure S17. Effect of light intensity for the photocatalytic degradation of AM dye in the presence of SnP-BTB.

Figure S18. Negative ion mode ESI-MS spectrum of the AM dye degradation reaction by SnP-BTB after 40 min of visible light irradiation.

Figure S19. Possible intermediates for the AM dye degradation in the presence of SnP-BTB after 40 min of visible light irradiation.

Figure S20. Band gap energy of SnP-BTB, SnP-BTC, and SnP has been calculated from the Tauc's Plot using absorption spectral data.

Figure S21. Photocurrent responses for SnP-BTB, SnP-BTC, and SnP under visible light.

Figure S22. EIS Nyquist plots for SnP-BTB, SnP-BTC, and SnP under visible light.

Figure S23. Visible light AM dye degradation activities of SnP-BTB in the presence of various scavengers.

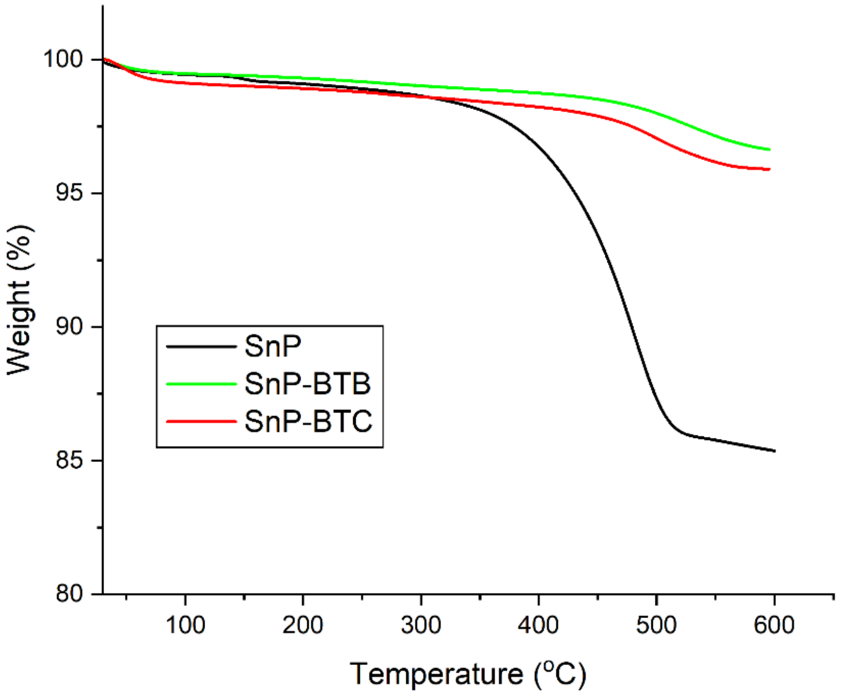

Figure S1. TGA thermogram of SnP, SnP-BTC, and SnP-BTB.

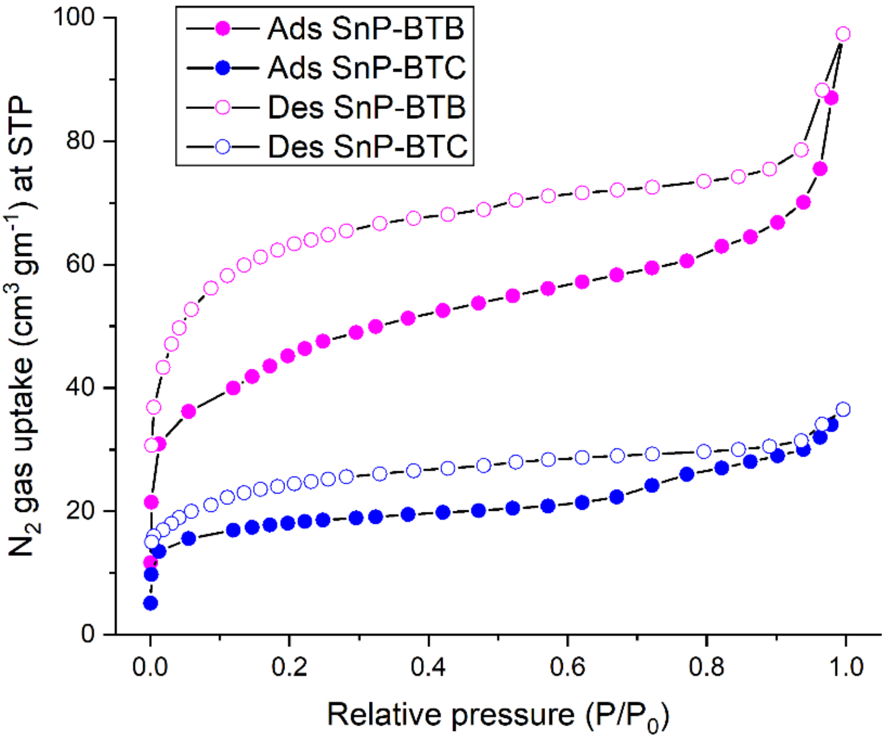

Figure S2. Adsorption and desorption isotherms of N_2 for SnP-BTC and SnP-BTB at 77 K.

(a)

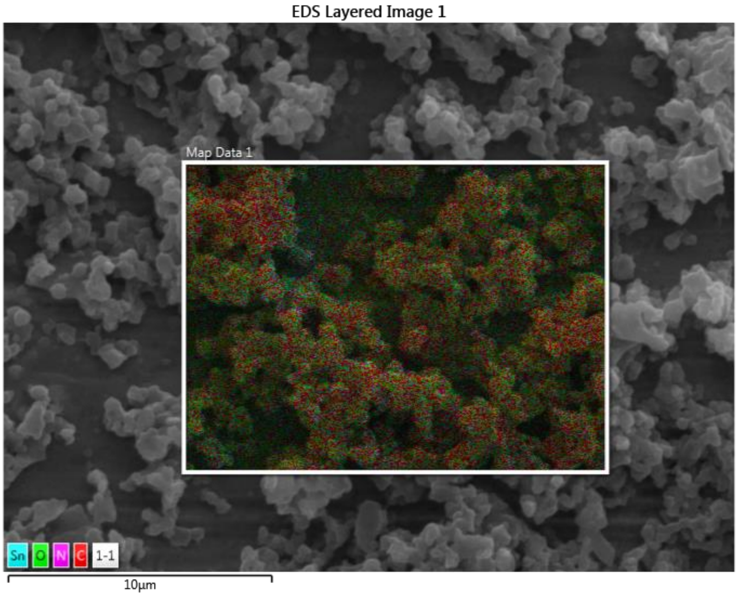

(b)

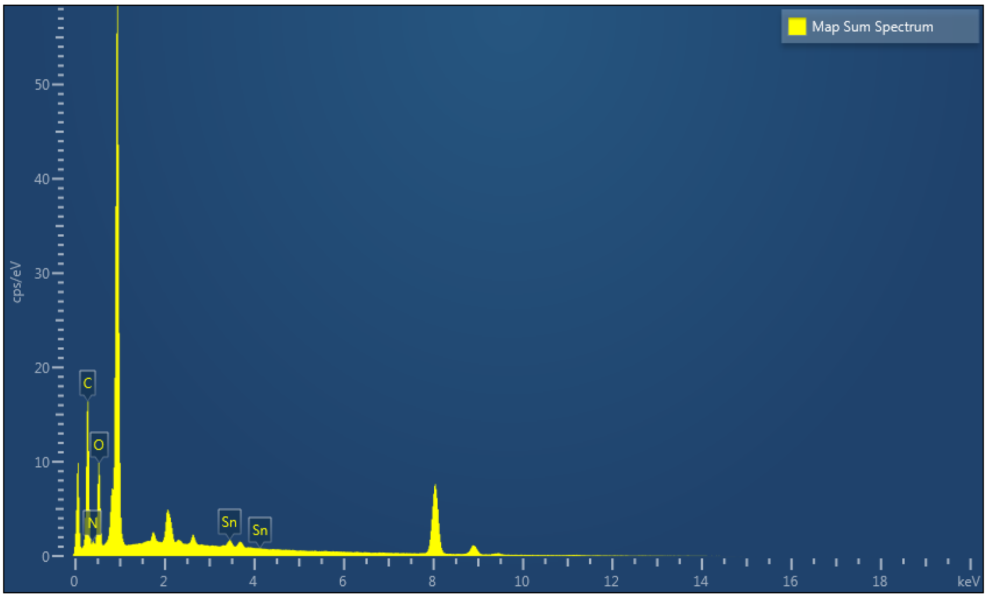

(c)

Element	Wt%	Atomic %
C	71.23	84.35
N	6.59	6.68
O	8.16	7.25
Sn	14.02	1.72
Total:	100.00	100.00

Figure S3. Energy dispersive X-ray spectroscopy (EDS) of SnP-BTC. Elemental mapping (a), spectra (b), table (c).

(a)

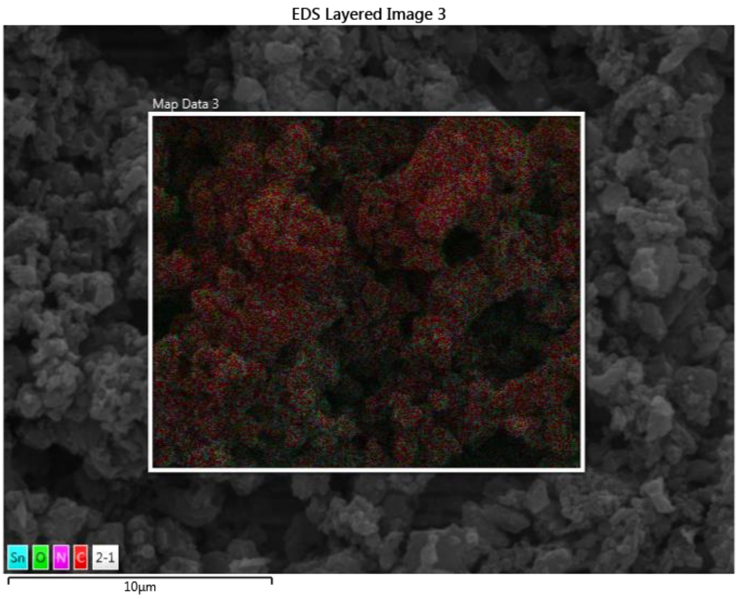

(b)

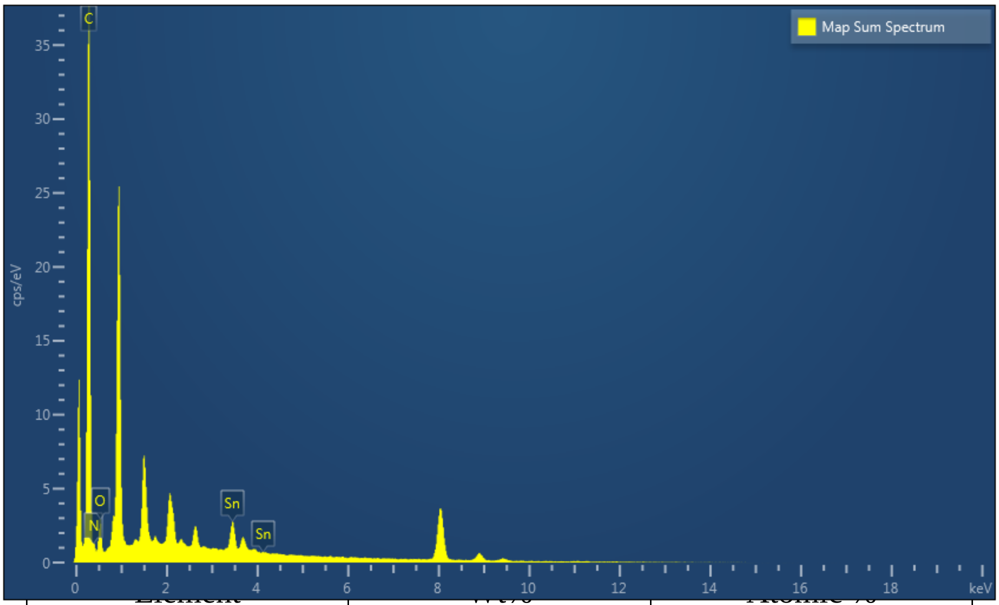

(c)

[illegible]	[illegible]	[illegible]
C	75.47	86.98
N	5.62	5.54
O	6.93	5.95
Sn	11.98	1.53
Total:	100.00	100.00

Figure S4. Energy dispersive X-ray spectroscopy (EDS) of SnP-BTB. Elemental mapping (a), spectra (b), table (c).

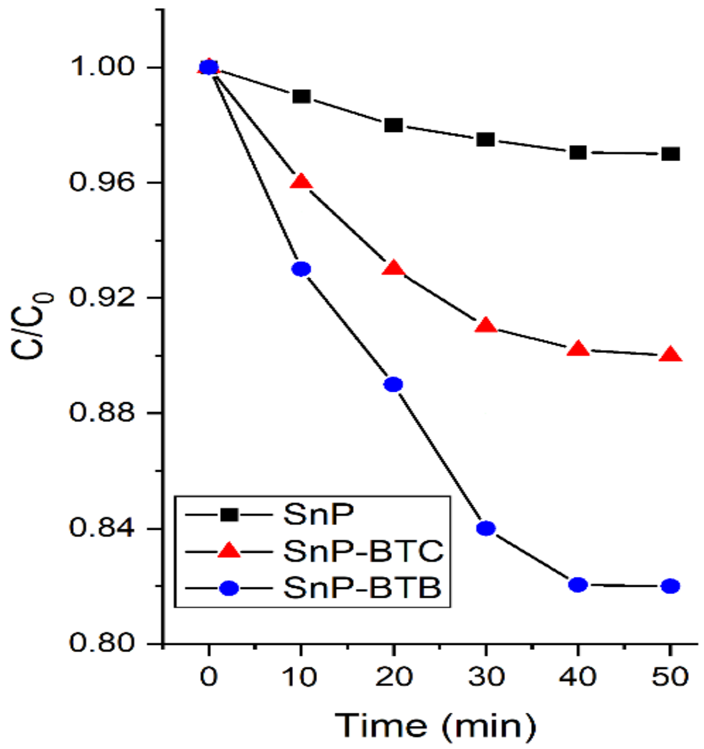

Figure S5. AM dye adsorption test of SnP, SnP-BTC, and SnP-BTB.

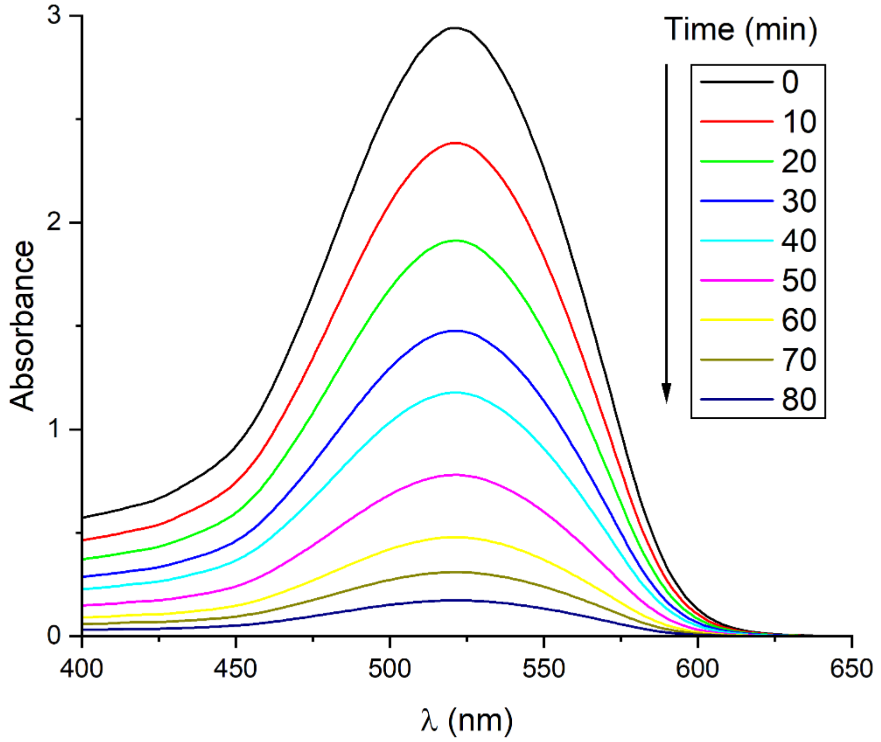

Figure S6. Absorption spectra of AM dye in the presence of SnP-BTB under visible light irradiation.

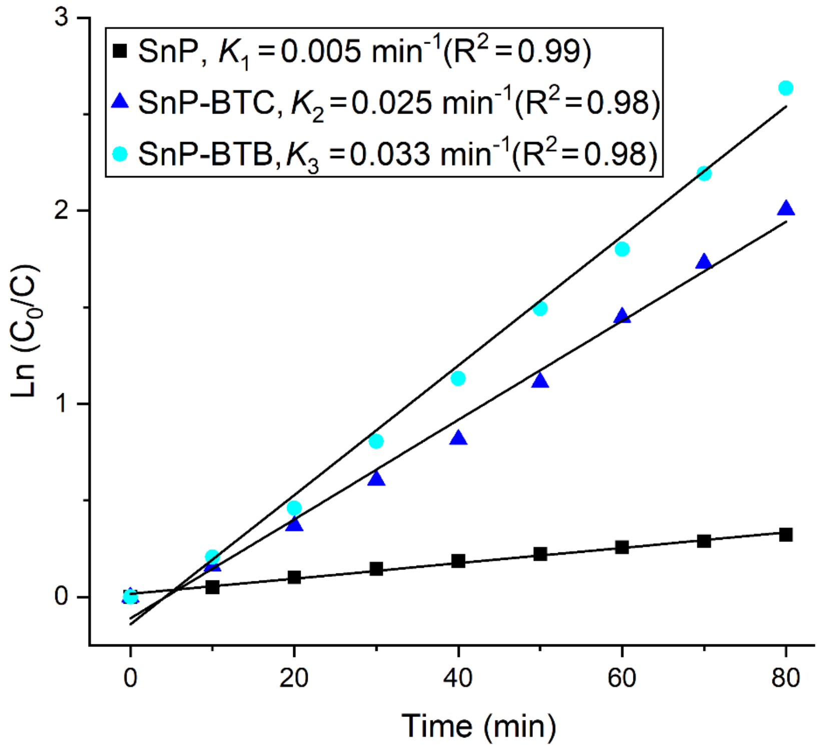

Figure S7. Kinetics of the photocatalytic degradation of AM under visible light irradiation.

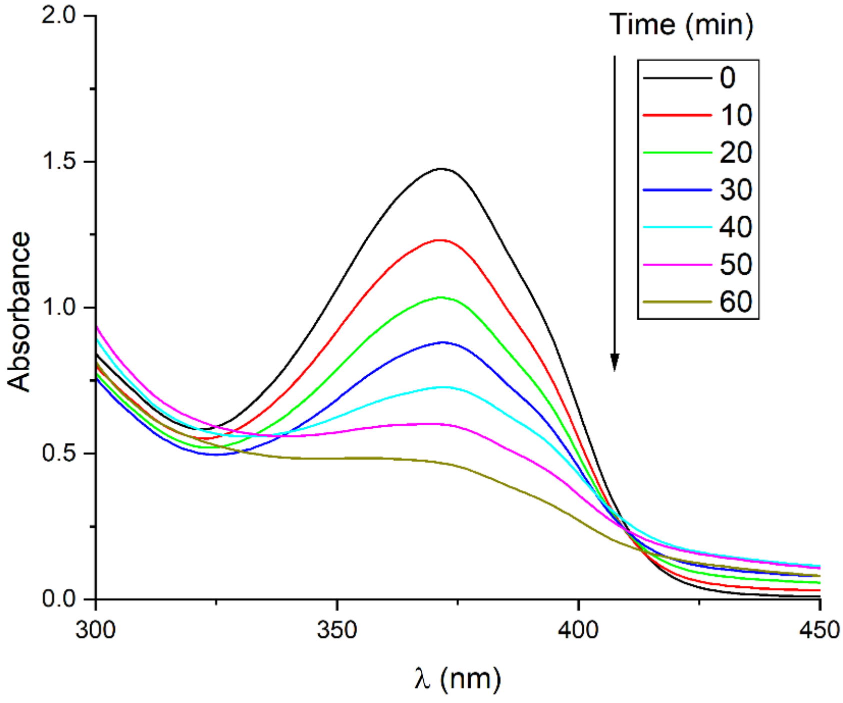

Figure S8. Absorption spectra of TC in the presence of SnP-BTB under visible light irradiation.

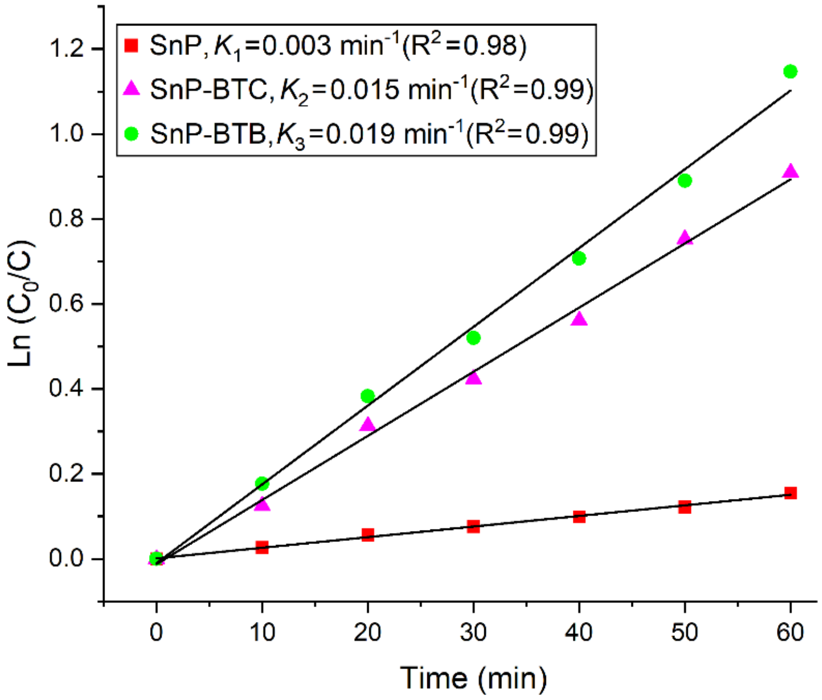

Figure S9. Kinetics of the photocatalytic degradation of TC under visible light irradiation.

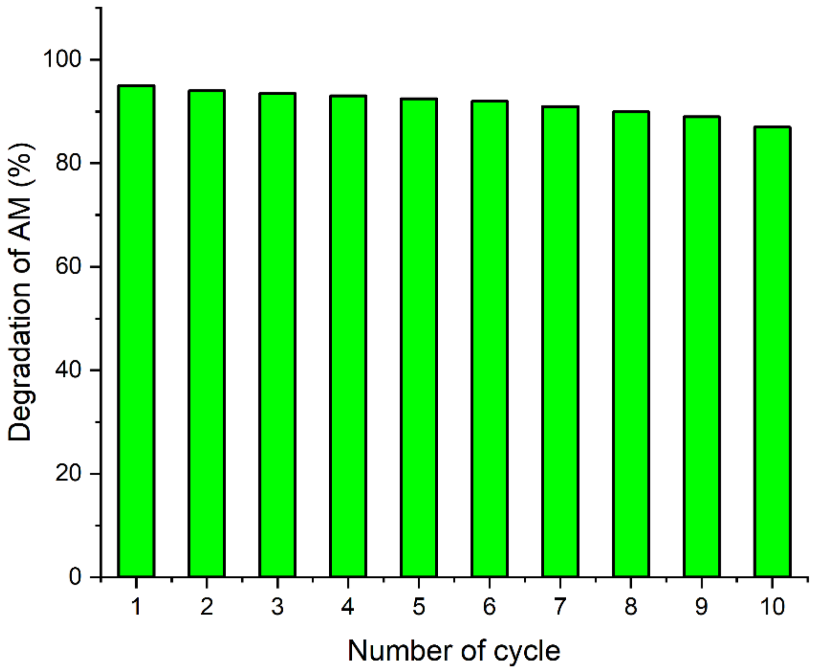

Figure S10. Recyclability of the photocatalyst SnP-BTB towards the degradation of AM dye.

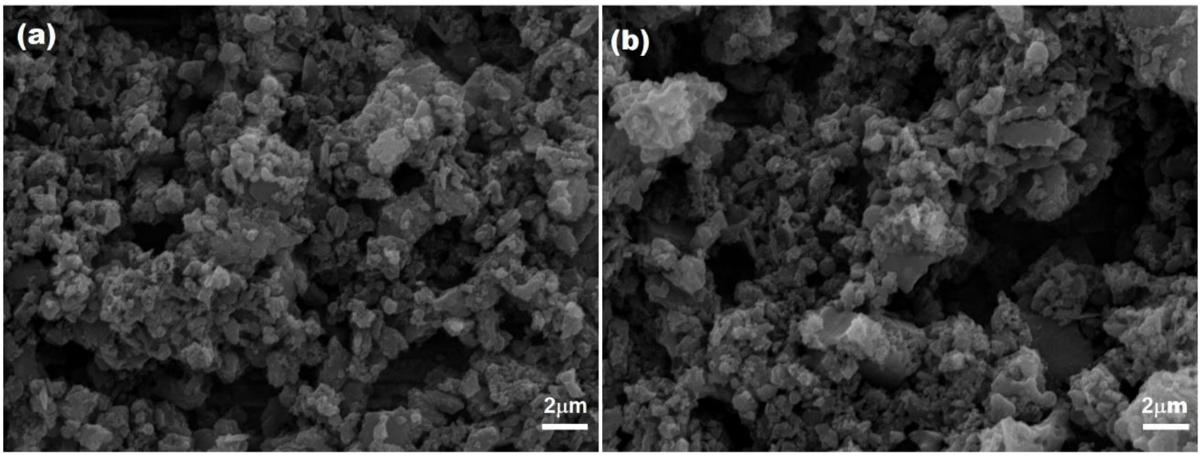

Figure S11. FE-SEM images of SnP-BTB after and before the degradation of AM dye). Fresh sample (a), and used sample (b).

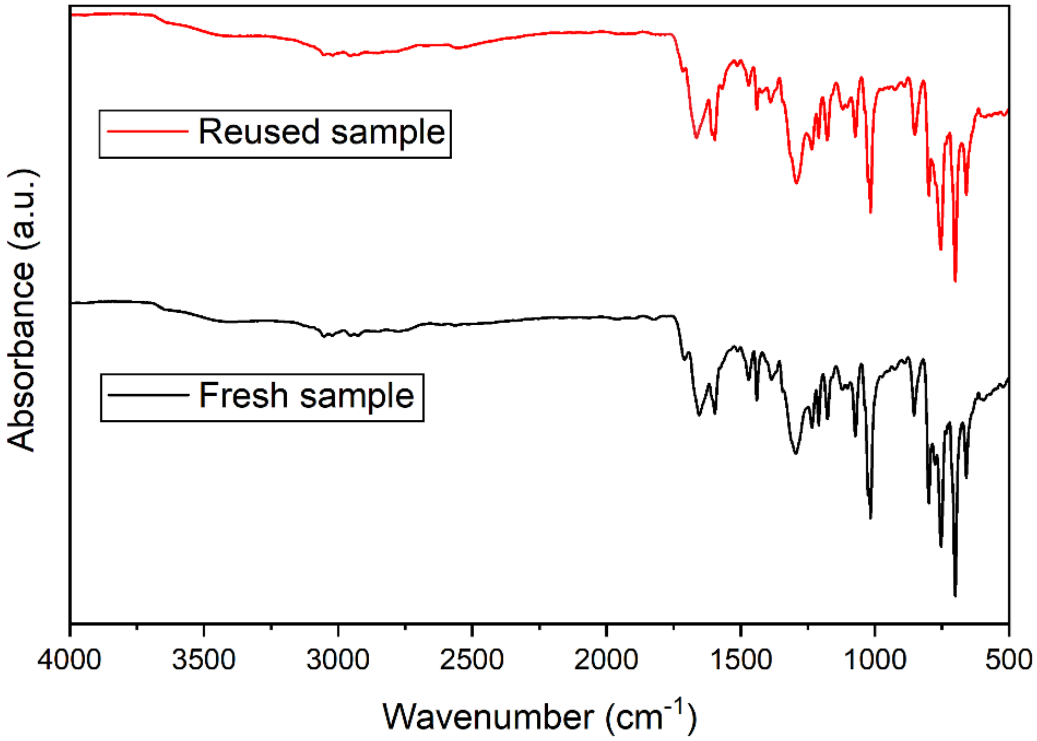

Figure S12. FT-IR spectra SnP-BTB (after and before the degradation of AM dye).

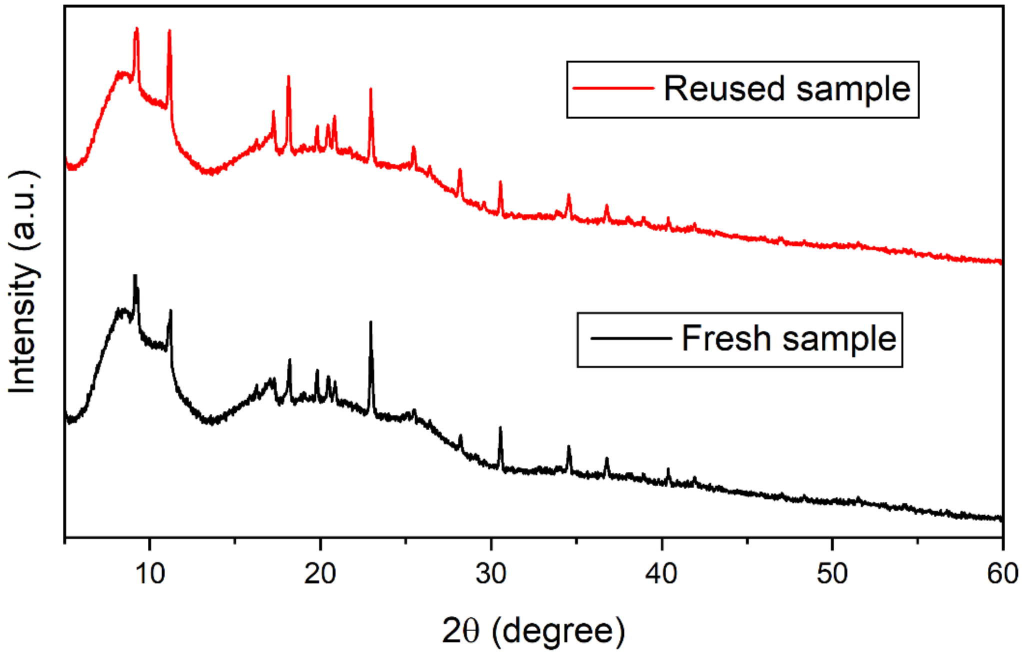

Figure S13. PXRD spectra SnP-BTB (after and before the degradation of AM dye).

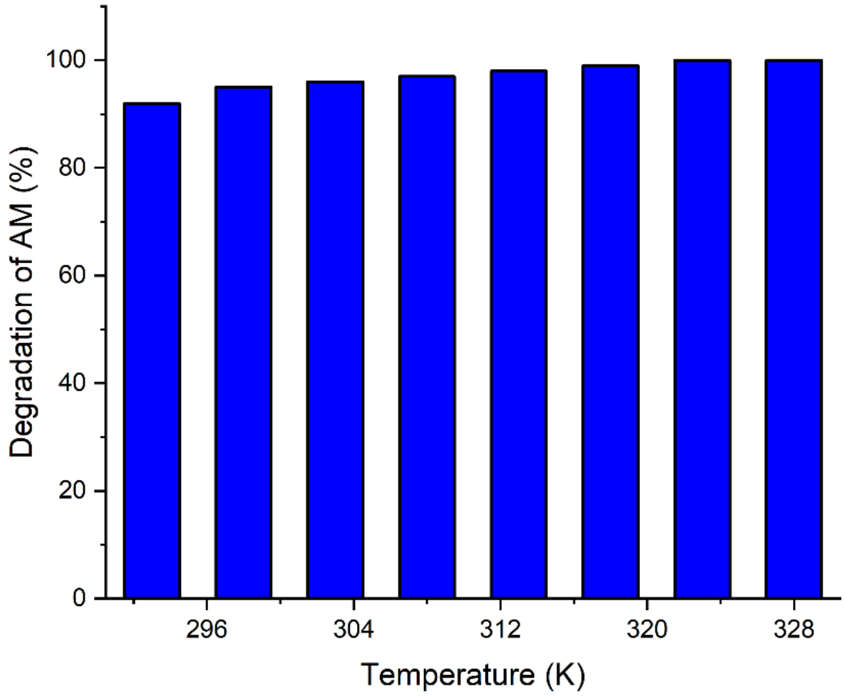

Figure S14. Effect of temperature for the photocatalytic degradation of AM dye in the presence of SnP-BTB.

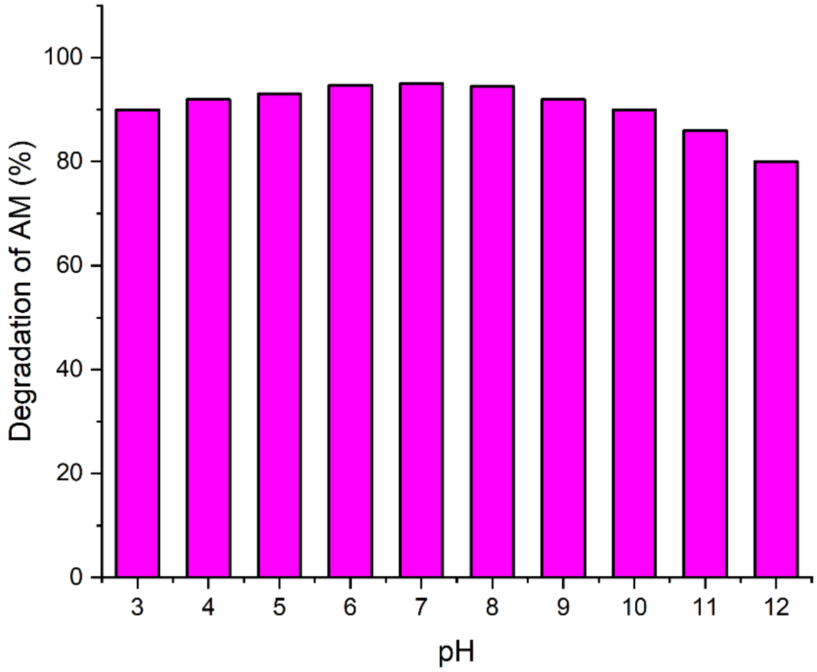

Figure S15. Effect of pH for the degradation of AM dye solution in the presence of SnP-BTB.

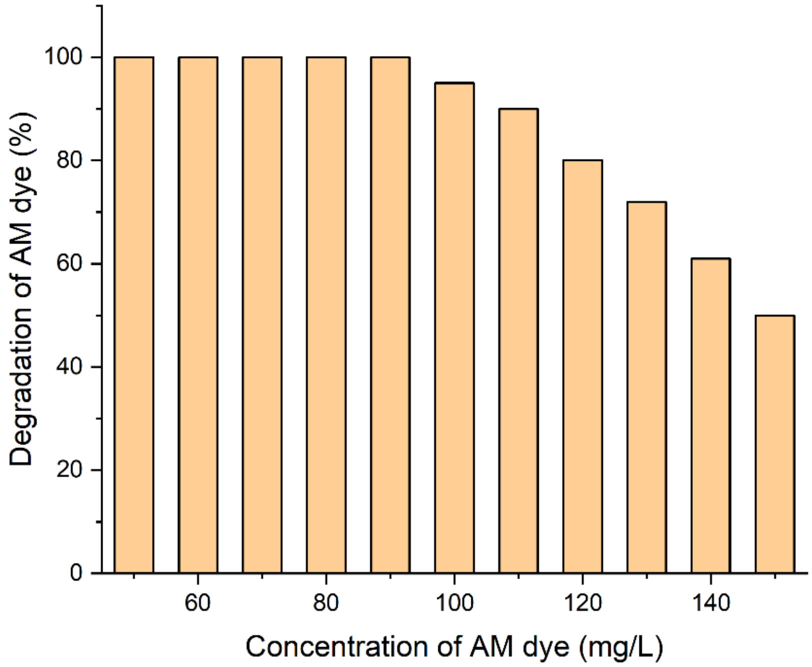

Figure S16. Effect of dye concentration for the photocatalytic degradation of AM dye in the presence of SnP-BTB.

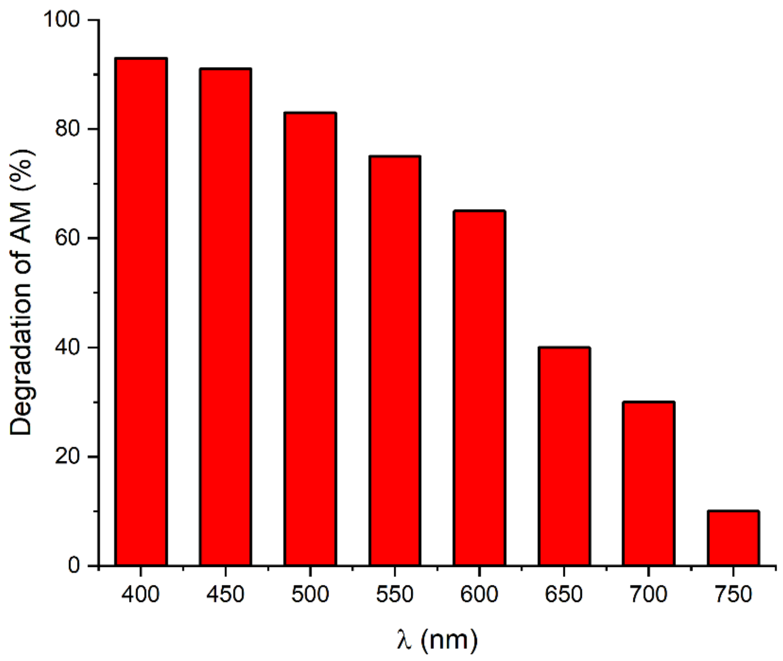

Figure S17. Effect of light intensity for the photocatalytic degradation of AM dye in the presence of SnP-BTB.

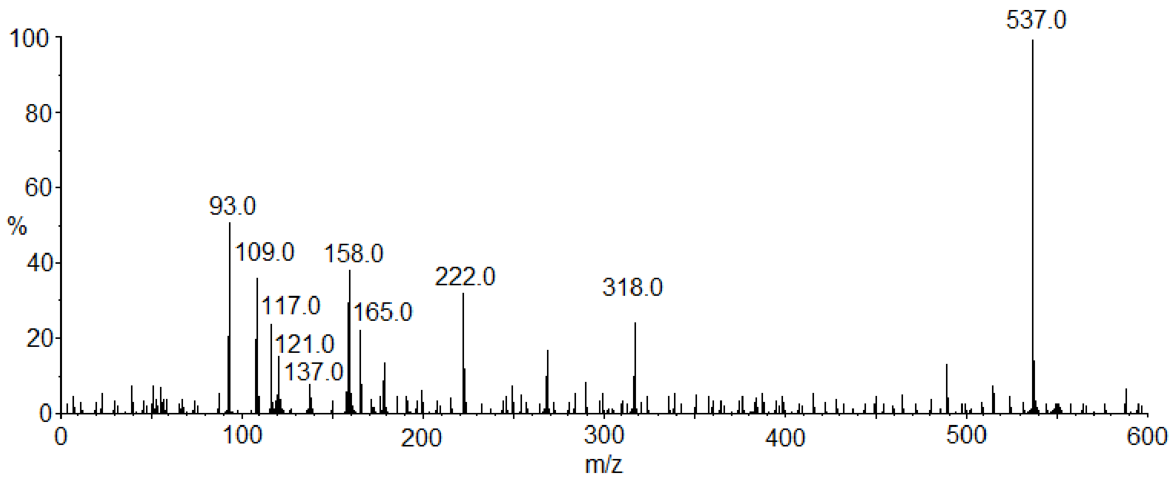

Figure S18. Negative ion mode ESI-MS spectrum of the AM dye degradation reaction by SnP-BTB after 40 min of visible light irradiation.

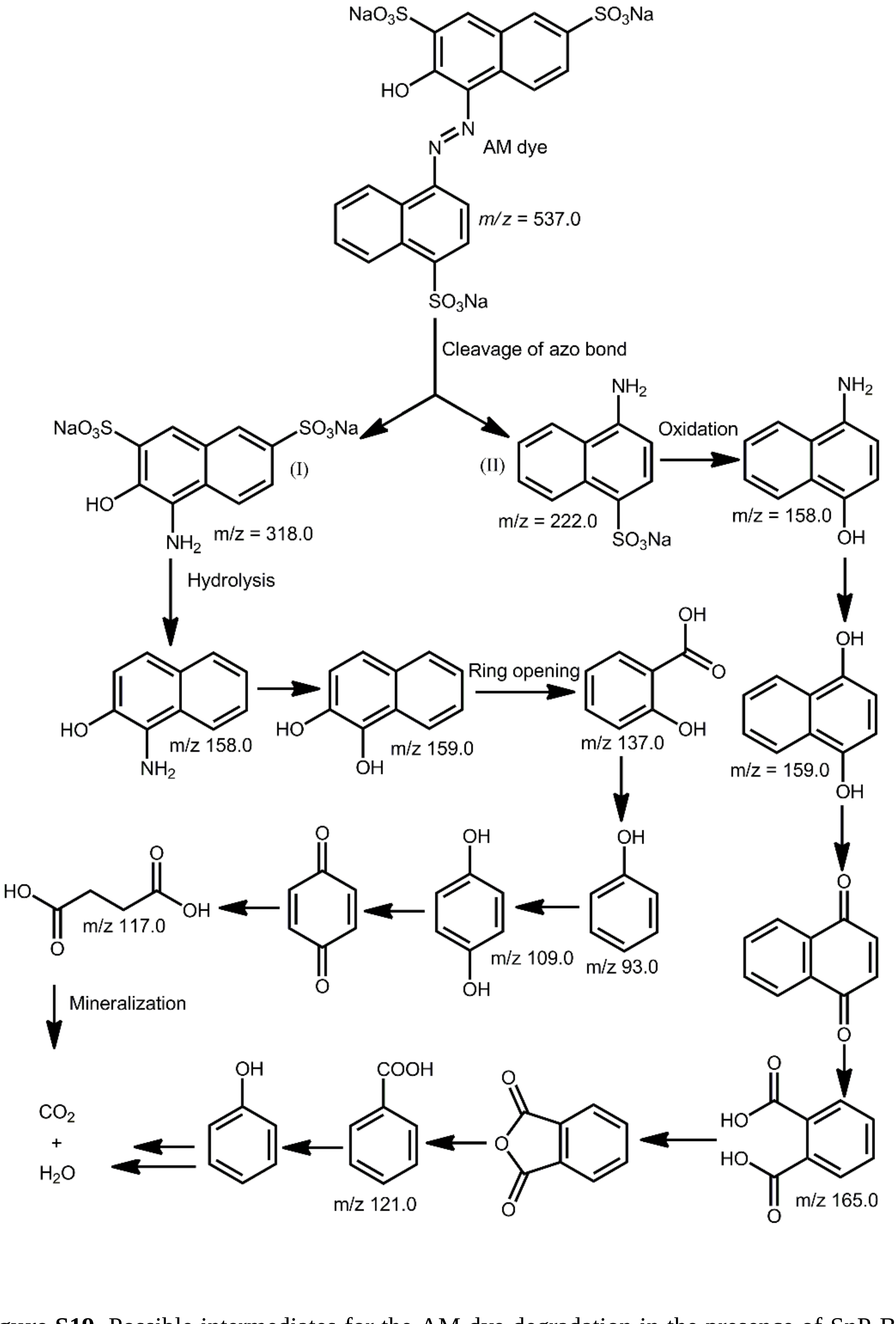

Figure S19. Possible intermediates for the AM dye degradation in the presence of SnP-BTB after 40 min of visible light irradiation.

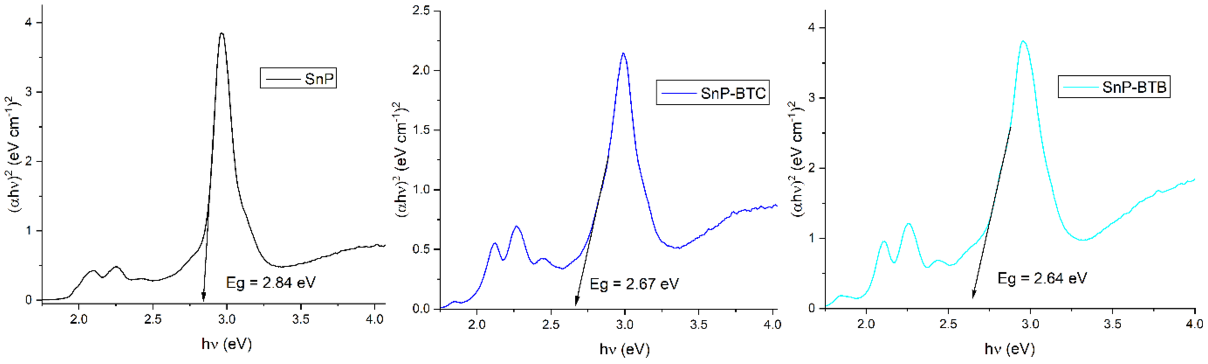

Figure S20. Band gap energy of SnP-BTB, SnP-BTC, and SnP has been calculated from the Tauc's Plot using absorption spectral data.

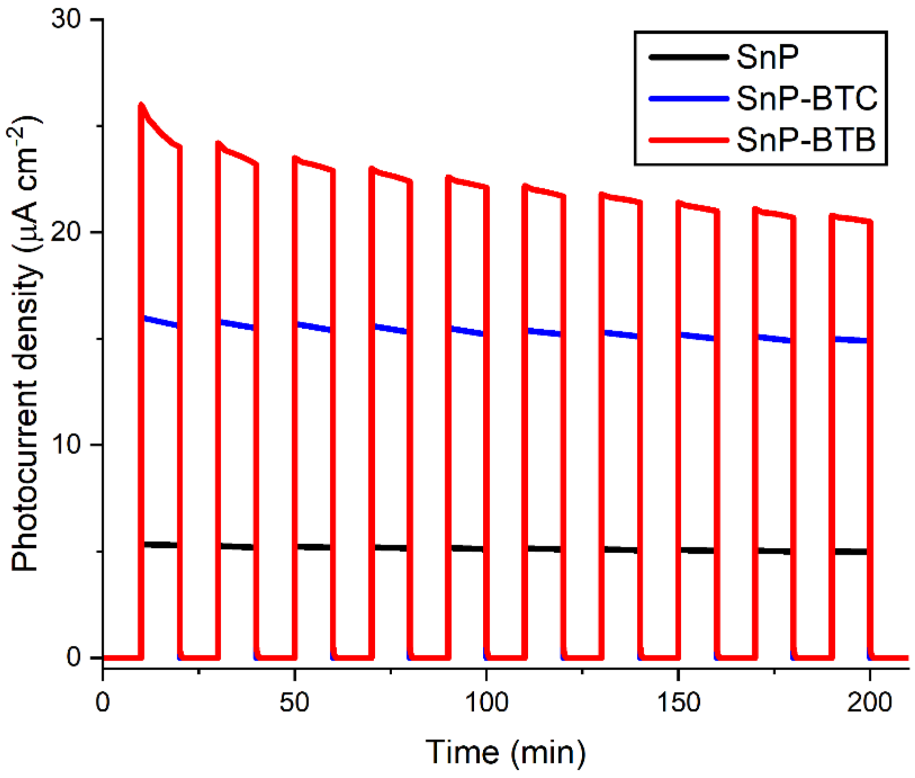

Figure S21. Photocurrent responses for SnP-BTB, SnP-BTC, and SnP under visible light.

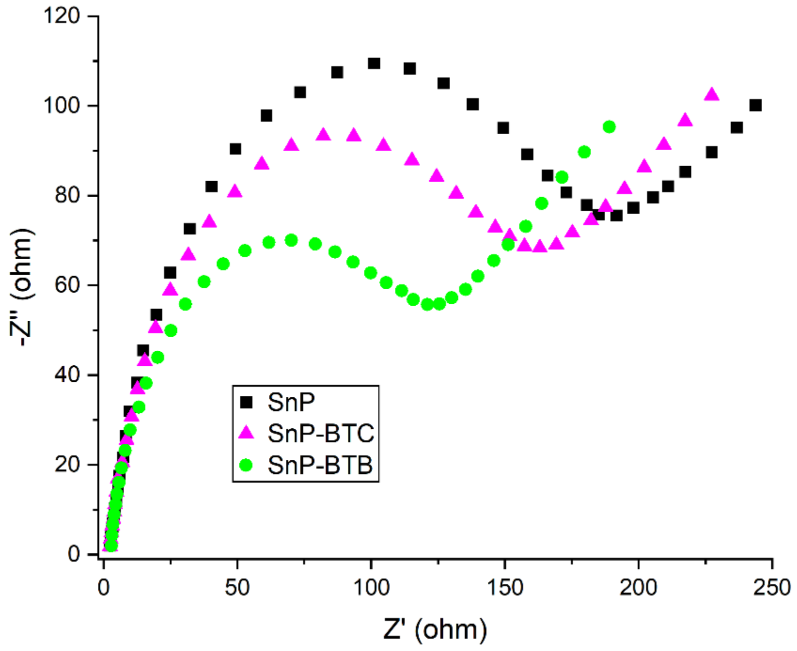

Figure S22. EIS Nyquist plots for SnP-BTB, SnP-BTC, and SnP under visible light.

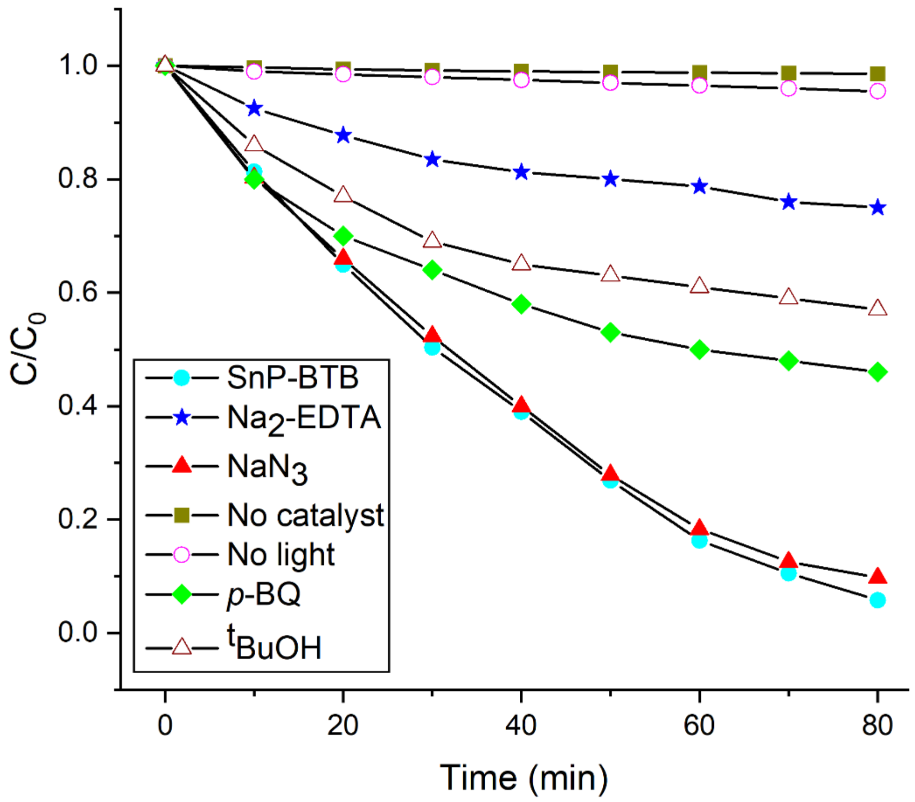

Figure S23. Visible light AM dye degradation activities of SnP-BTB in the presence of various scavengers.
